# Metabolome Analysis of *Arabidopsis thaliana* Roots Identifies a Key Metabolic Pathway for Iron Acquisition

**DOI:** 10.1371/journal.pone.0102444

**Published:** 2014-07-24

**Authors:** Holger Schmidt, Carmen Günther, Michael Weber, Cornelia Spörlein, Sebastian Loscher, Christoph Böttcher, Rainer Schobert, Stephan Clemens

**Affiliations:** 1 Department of Plant Physiology, University of Bayreuth, Bayreuth, Germany; 2 Department of Organic Chemistry, University of Bayreuth, Bayreuth, Germany; 3 Department of Stress and Developmental Biology, Leibniz Institute of Plant Biochemistry, Halle/Saale, Germany; 4 Bayreuth Center for Molecular Biosciences, University of Bayreuth, Bayreuth, Germany; Wake Forest University, United States of America

## Abstract

Fe deficiency compromises both human health and plant productivity. Thus, it is important to understand plant Fe acquisition strategies for the development of crop plants which are more Fe-efficient under Fe-limited conditions, such as alkaline soils, and have higher Fe density in their edible tissues. Root secretion of phenolic compounds has long been hypothesized to be a component of the reduction strategy of Fe acquisition in non-graminaceous plants. We therefore subjected roots of *Arabidopsis thaliana* plants grown under Fe-replete and Fe-deplete conditions to comprehensive metabolome analysis by gas chromatography-mass spectrometry and ultra-pressure liquid chromatography electrospray ionization quadrupole time-of-flight mass spectrometry. Scopoletin and other coumarins were found among the metabolites showing the strongest response to two different Fe-limited conditions, the cultivation in Fe-free medium and in medium with an alkaline pH. A coumarin biosynthesis mutant defective in *ortho*-hydroxylation of cinnamic acids was unable to grow on alkaline soil in the absence of Fe fertilization. Co-cultivation with wild-type plants partially rescued the Fe deficiency phenotype indicating a contribution of extracellular coumarins to Fe solubilization. Indeed, coumarins were detected in root exudates of wild-type plants. Direct infusion mass spectrometry as well as UV/vis spectroscopy indicated that coumarins are acting both as reductants of Fe(III) and as ligands of Fe(II).

## Introduction

Virtually all life forms depend on iron (Fe) for basic cell function, growth and development. The availability of its ferric (Fe^3+^) and ferrous (Fe^2+^) oxidation states under physiological conditions make the metal essential for a plethora of electron transfer reactions, e.g. in photosynthesis and respiration [1], [2]. Consequently, Fe deficiency results in a number of adverse effects for organisms. In humans, undersupply with this micronutrient is the most widespread nutritional disorder in the world. Fe deficiency anemia is estimated to affect about 30% of the world’s population. It substantially increases the risk of maternal and perinatal mortality and can be the cause of impaired mental development [3]. Since a large fraction of the human diet is plant-based, there is an urgent need for crops that more efficiently accumulate Fe in a bioavailable form in their edible parts. Prerequisite for this biofortification approach is the understanding of Fe uptake into and its distribution within plants for breeding or engineering new varieties [4], [5].

Fe acquisition poses a major challenge. While the metal is one of the most prevalent elements in the Earth’s crust and highly abundant in soils, it is not readily available for plant uptake in the presence of oxygen as it precipitates in the form of insoluble ferric oxides. This process is strongly enhanced at alkaline pH with dramatic consequences for plant productivity especially on calcareous soils. It is estimated that 30% of the world’s arable land is too alkaline for optimal plant growth [6].

Plants have evolved two different strategies for Fe acquisition, the acidification-reduction strategy (strategy I) and the chelation strategy (strategy II) [2], [7]. The latter is used by grasses. It is based on the solubilization of ferric precipitates through the secretion of strong Fe(III)-chelating molecules. These phytosiderophores of the mugineic acid family are released into the rhizosphere by efflux transporters such as TOM1 [8]. Uptake of Fe(III)-phytosiderophore complexes into the symplast is mediated by YS1/YSL-transporters [9]. Non-graminaceous plants acquire Fe by a combination of rhizosphere acidification, the extracellular reduction of ferric to ferrous iron at the root surface, and subsequent uptake of ferrous iron into epidermal cells. The proteins mainly responsible for these processes in *A. thaliana* are AHA2, FRO2 and IRT1, respectively [10]–[12].

A major gap in our understanding of strategy I has been the potential role of phenolic compounds secreted by the roots [6]. The release of small molecules into the rhizosphere is an important component of a plant’s response to fluctuations in the abiotic and biotic environment [13]. Root exudates can, for instance, function as allelochemicals or alter the surrounding substrate. It is well documented that phenolic compounds including flavonoids comprise a large fraction of the root exudates of strategy I plants under Fe deficiency [14]. They have been hypothesized to mobilize apoplastically bound Fe(III) for uptake into the symplast. Studies on red clover showed that removal of secreted phenols from the roots led to Fe deficiency of the shoot even though acidification and ferric reductase activity were enhanced. These observations suggested a crucial role for secreted phenols in Fe acquisition independent of established strategy I processes [15]. In rice, which is exceptional as it employs both strategy I and strategy II [2], the efflux transporter PEZ1 was recently described as an integral component of strategy I [16]. It mediates the secretion of compounds such as caffeic acid and is essential for the solubilization of apoplastic Fe oxides.

The exact mechanisms underlying the mobilization of Fe(III) are unknown. Phenolic compounds including flavonoids have been discussed both as chelators of Fe(III) and as reductants [14], [17], [18]. Until recently, neither specific information on the activity of particular compounds nor direct genetic evidence for the importance of phenol secretion in Fe acquisition by non-graminaceous plants was available. Rodriguez-Celma et al. then showed that secretion of phenolics is critical for *A. thaliana* Fe acquisition from low bioavailability sources [19]. Later it was demonstrated through the analysis of mutants defective in the ABC transporter ABCG37 [20] and in coumarin biosynthesis [21], respectively, that the active compounds are coumarins.

Our objective was to identify molecules involved in the acquisition of Fe by strategy I plants through a metabolomics approach. We subjected roots of *A. thaliana* plants grown under Fe-replete and two different Fe-deplete conditions to comprehensive metabolome analysis by gas chromatography-mass spectrometry (GC-MS) as well as ultra-pressure liquid chromatography electrospray ionization quadrupole time-of-flight mass spectrometry (UPLC-ESI-QTOF-MS) and searched for compounds increasing in abundance under Fe deficiency. Several hundred features (*m/z* – retention time pairs) showing significant differences were detected. Among the most prominent changes various coumarins could be identified. A respective biosynthetic mutant was unable to grow on alkaline soil unless treated with extra Fe. Co-cultivation with wild type plants partially rescued the mutant phenotype. Taken together, our data clearly confirm the secretion of coumarins as an essential component of *A. thaliana* Fe acquisition and provide more extensive information on metabolome changes elicited by Fe deficiency. The latter can guide future studies aiming at a systems level understanding of plant responses to Fe-deplete conditions.

## Materials and Methods

### Plant material

*Arabidopsis thaliana* accession Columbia (Col-0) was used as wild type background (wt). T-DNA insertion lines *f6*′*h1-1* and *f6*′*h1-5* (SALK_132418c and SALK_050137) were obtained from the Nottingham Arabidopsis Stock Centre (University of Nottingham, UK) [22]. The *f6*′*h1-1* mutant carries a T-DNA insertion in exon 1 of At3g13610 and was described before as coumarin-deficient [23]. The *f6*′*h1-5* line carries a T-DNA insertion in exon 2. This was confirmed by genotyping (File S1). Plants were cultivated in a growth cabinet (Percival Scientific) at a temperature cycle of 23°C (day)/18°C (night) and a photoperiod of 8 h light (110 µE m^−2^s^−1^) and 16 h dark. Plants were grown on normal soil or on soil that was alkalinized by addition of CaO resulting in a pH of 7.7. Fe fertilization of alkaline soil was performed by watering with 0.5% Fetrilon, which contains Fe-EDTA (BASF, Germany). For hydroponic culture, 1/10 Hoagland Solution 2 (0.28 mM Ca(NO_3_)_2_, 0.6 mM KNO_3_, 0.1 mM NH_4_H_2_PO_4_, 0.2 mM MgSO_4_, 4.63 µM H_3_BO_3_, 32 nM CuSO_4_, 915 nM MnCl_2_, 77 nM ZnSO_4_, 11 nM MoO_3_) with or without Fe-HBED (5 µM) as Fe source at pH 5.7 was used [24], [25]. Seeds were germinated on solid medium-filled PCR tubes. Ten days after germination, plants were transferred to cultivation vessels with liquid medium. We either used 50 ml Falcon tubes for cultivation of single plants or 1.6 l pots for cultivation of three plants. Fe limitation was imposed in two different ways. Fe-free medium was prepared by omitting Fe-HBED. For alkaline growth conditions, medium was buffered with 10 mM NaHCO_3_ and adjusted with KOH to pH 7.7. Medium was changed weekly and pH was checked. The metabolome analysis is based on two sets of three independent experiments (medium +Fe vs. medium –Fe; medium pH 5.7 vs. medium 7.7) performed over a period of approximately fifteen months. Three plants were pooled for one sample. Roots were harvested and blotted dry with paper towels, flash-frozen in liquid nitrogen and stored at −80°C. Prior to metabolite extraction root tissue was ground to a fine powder in liquid nitrogen.

### Collection of exudates

Exudates were collected from hydroponically grown plants (50 ml tubes) through transfer into 50 ml distilled water for 24 h. Samples were freeze-dried and the metabolites re-dissolved in 0.5 ml 80% methanol per tube. Pooled exudates from 45 plants were centrifuged and 5 µl were analyzed as described for the root extracts. Three independent experiments were performed and analyzed.

### Elemental analysis via inductively coupled plasma-optical emission spectroscopy (ICP-OES)

Root and leaf material was freeze-dried and digested in 4 ml 65% HNO_3_ and 2 ml 30% H_2_O_2_ using a microwave (START 1500, MLS GmbH). Fe concentrations were measured with an iCAP 6500 (Thermo Scientific).

### Metabolite profiling by GC-MS

For GC-MS measurements, metabolites were extracted and samples derivatized as described [26]. The GC-MS setup consisted of a Gerstel MPS autosampler, an Agilent 7890A GC system and a 5975C inert MSD (Gerstel GmbH, Mülheim/Ruhr, Germany). Chromatographic separation was performed on a Zebron Guardian ZB-5 (40 m×0.25 mm, 25 µm, 10 m integrated precolumn, Phenomenex Aschaffenburg, Germany). Data were analyzed via AMDIS (Ver. 2.71) and SpectConnect using the default settings of the respective software [27]–[30]. The resulting SpectConnect matrix was imported into Microsoft Excel. Entries were normalized to the internal standard ribitol and filtered according to their mean fold-change (<0.66 or >1.5) and P value (<0.05) in a Student’s t-test. Only entities above a relative abundance of 1% of the ribitol peak were further characterized. A compound was regarded as identified when matched to the in house library (ΔRI<10, R-match >800).

### Metabolite profiling by UPLC-ESI-QTOF-MS

A Waters Aquity UPLC system equipped with a HSS T3 column (1.8 µm, 2.1×100 mm) coupled to a Q-TOF Premier mass spectrometer was used for metabolite profiling (Waters Corporation, Milford, MA). Extraction and analysis of plant metabolites was essentially performed as described [31], [32]. A linear gradient at a flow of 0.5 ml·min^−1^ from water (A) to acetonitrile (B), both acidified with 0.1% formic acid, was applied as follows: 0 min 99.5% A, 1 min 99.5% A, 16 min 8.5% A, 19 min 0.5% A, 22 min 0.5% A, 23 min 99.5% A, 24 min 99.5% A. Raw data were converted to the netCDF format prior to analysis and visualization via XCMS online [33]–[35]. MS2Ts of pooled root samples were recorded as described [36], [37]. The recorded MSMS spectra were compared to the primeE-MS2T database and Massbank [38].

### Direct infusion mass spectrometry

To study the interaction of ferrous and ferric iron with coumarins, 10 mM aqueous solutions of FeCl_3_ and FeCl_2_ were prepared. Methanolic solutions (10 mM) of the ligands esculetin, fraxetin and scopoletin were added in different stoichiometric ratios to an aqueous solution of the respective iron salt. The final Fe concentration in solution was 1 mM. The mixtures (Fe:ligand ratio varying from 1∶1 to 1∶3) were injected into the mass spectrometer via a syringe pump. Source settings corresponded to those used for LC-MS analysis and flow rate was adjusted to achieve maximum signal intensities. Collision energy in MS-MS experiments was set to 15 V.

### Spectral analysis of Fe-coumarin complexes

We prepared, under exclusion of air and moisture, acetonitrile solutions that contained three equivalents of any one of these ligands, six equivalents of the base triethylamine, and one equivalent of either FeCl_2_ or FeCl_3_. After stirring for 30 min the solutions were transferred under argon atmosphere to a UV cuvette and the optical spectra were recorded at wavelengths ranging from 300 to 900 nm.

### Overexpression of F6′H1

For the complementation of *f6*′*h1-5* the *F6*′*H1* coding sequence (cds) was amplified from root cDNA (F6-H1-cds-fw: 5′-atggctccaacactcttgacaacc-3′; F6-H1-cds-wo-Stop-rev: 5′-gatcttggcgtaatcgacggtt-3′) and cloned into pCR8/GW/TOPO (Invitrogen Life Technologies). Using gateway technology (Invitrogen Life Technologies) the cds was subcloned into pMDC83 which adds a C-terminal GFP-tag [39]. Both cloning steps were performed according to the manufacturer’s instructions. The final construct was Agro-transformed into SALK_050137 (*f6*′*h1–5*) by the floral dip method.

### Chemicals

Standards of fraxin and scopolin were obtained from Phytolab GmbH & Co. KG (Vestenbergsgreuth, Germany). Scopoletin was synthesized based on Demyttenaere et al. [40], [41]. All other standards were obtained from Sigma-Aldrich (Taufkirchen, Germany).

## Results

**Figure 1:**
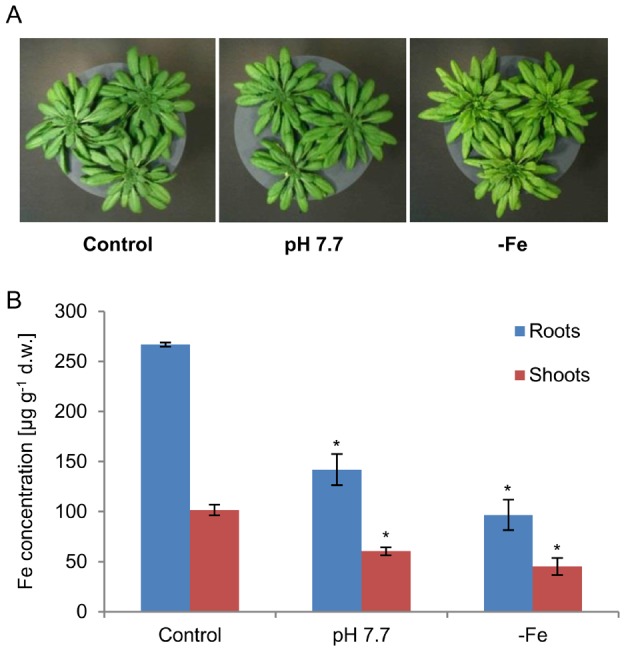
*A. thaliana* Col-0 plants were exposed to two different conditions causing Fe deficiency. (A) Plants were grown hydroponically in 1/10 Hoagland Solution for six weeks. Plants were cultivated either at a pH of 5.7 with Fe-HBED as Fe source (Control), at a pH of 7.7 with Fe-HBED as Fe source (pH 7.7), or the final two weeks at a pH of 5.7 without Fe-HBED (−Fe). (B) Fe concentrations in roots (blue bars) and shoots (red bars) were determined by ICP-OES. The means of three independent biological experiments are displayed. Error bars indicate standard deviation. Significant differences to plants grown under control conditions were determined by Student’s t-test, *P<0.05.

*A. thaliana* Col-0 wild type plants were cultivated hydroponically for easy access to root tissue. In order to allow stringent filtering of the highly complex metabolomics data, we imposed Fe deficiency conditions in two different ways in separate sets of three independent experiments each. First, four week old plants were grown for additional two weeks in medium without added Fe-HBED. Second, plants were cultivated for six weeks in medium with the pH adjusted to 7.7 (instead of 5.7). Elemental profiles of roots and shoots were determined by ICP-OES. Both the omission of Fe for two weeks and a medium pH of 7.7 resulted in significant reductions of root and shoot Fe concentrations indicating Fe deficiency (Fig. 1).

### GC-MS metabolite profiling

**Figure 2:**
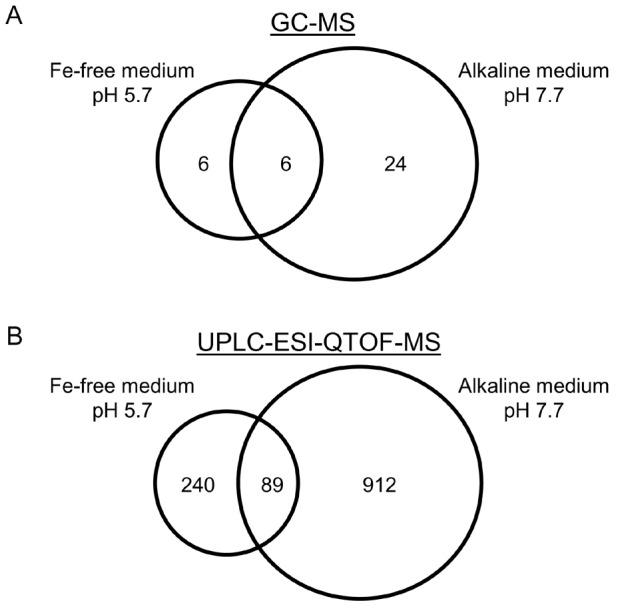
The root metabolome changes triggered by two different conditions of Fe deficiency partially overlap. Venn diagrams display the total number of significant metabolic changes detected by GC-MS (A) and UPLC-ESI-QTOF-MS (B) when comparing growth in the presence of Fe with growth in Fe-free medium for two weeks, or growth at pH 5.7 with growth at pH 7.7. Detailed analysis focused on the metabolites and features (*m/z* – retention time pairs) that were shared by the two conditions of Fe deficiency (see Tables 1 and 2, Tables S1 and S2 in File S6).

**Figure 3:**
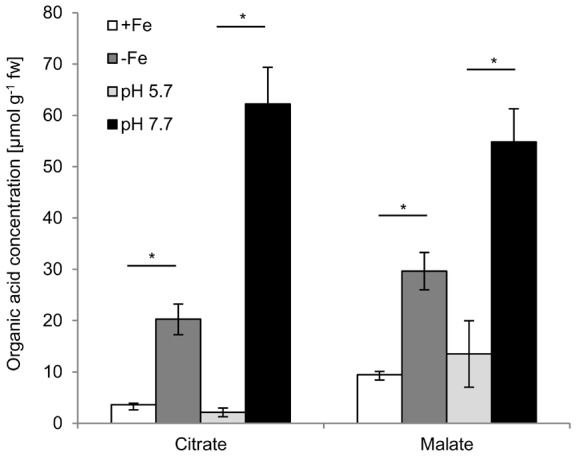
Citrate and malate showed strong increases of root concentrations in response to Fe deficiency. *A. thaliana* Col-0 plants were cultivated hydroponically and in separate sets of experiments exposed to two different conditions of Fe deficiency. First, root metabolomes of plants cultivated in control conditions (+Fe, white bars) were compared to those grown in Fe-free medium for two weeks (−Fe, dark grey bars). Second, plants were cultivated at pH 5.7 (light grey bars) and at pH 7.7 (black bars). Shown is the quantification of citrate and malate, the most prominent changes detected by GC-MS (see Tables 1 and 2). The means of three independent biological experiments are displayed. Error bars indicate standard deviation. Significant differences between respective control and Fe deficiency conditions were determined by Student’s t-test, *P<0.05.

GC-MS analysis of root extracts and relative quantification detected in total 12 significant changes (>1.5fold, P<0.05) in response to Fe-free conditions and 30 in response to alkaline conditions (Fig. 2A; Tables 1 and 2). The two dominant metabolic changes in plants grown in Fe-free medium relative to plants grown in control medium were increases in the abundance of the organic acids citrate and malate. Quantification based on standard curves showed 5.6-fold and 3.1-fold higher concentrations, respectively (Fig. 3). Similarly, in root metabolite profiles of plants grown in alkaline medium, signals for these two compounds were again the most prominent changes (Table 2). Concentrations were 29.3-fold (citrate) and 4.1-fold (malate) higher than in profiles of plants grown under control conditions (Fig. 3).

**Table 1:** Metabolic changes in *A. thaliana* roots upon cultivation in hydroponic medium without added Fe as identified by GC-MS metabolite profiling.

Metabolite	relative abundance −Fe	relative abundance +Fe	fold change	P-value
Citric acid	0.72±0.3	0.13±0.03	5.6	<0.001
Malic acid	0.65±0.36	0.21±0.06	3.1	0.006
Fructose	0.08±0.05	0.2±0.09	0.4	0.005
Alanine	0.05±0.04	0.08±0.03	0.6	0.048
Asparagine	0.05±0.02	0.02±0.01	3.1	0.002
Threonine	0.04±0.01	0.02±0.01	1.7	0
n. i. (Methyl-3-hydroxy-pentandioic acid, R-Match: 930, RI: 1405)	0.02±0.03	<0.01	28.6	0.034
Galactinol	<0.01	0.02±0.01	0.1	<0.001
n. i. (Aspartic acid, R-Match: 861, RI: 1537)	0.01±0	0.02±0.01	0.6	0.037
n. i. (Lysine, R-Match: 831; RI: 1945)	0.01±0.01	<0.01	n. d.	0.002
n. i. (2-Ketoglutaric acid, R-Match: 831, RI: 1579)	0.01±0.01	<0.01	4.3	0.025
n. i. (Phosphoric acid, R-Match: 833, RI: 1813)	0.01±0.01	<0.01	2.4	0.014

Polar metabolites were extracted with methanol, derivatized and subjected to GC-MS analysis. Listed are compounds that showed a significant (P<0.05, Student’s t-test) difference (>1.5-fold) in three independent biological experiments. Entries are ordered according to their maximum relative abundance under –Fe conditions; n. i.: not unambiguously identified (no comparison with authentic standard), RI: retention index, R-Match: reverse match (only values above 800 are given).

**Table 2:** Metabolic changes in *A. thaliana* roots upon cultivation in hydroponic medium with alkaline pH as identified by GC-MS metabolite profiling.

Metabolite	relative abundance pH7.7	relative abundance pH 5.7	fold change	P-value
Citric acid	2.22±0.26	0.08±0.06	31.8	<0.001
Malic acid	1.2±0.14	0.3±0.34	4.4	<0.001
Threonine	0.36±0.24	0.07±0.05	5.4	0.006
n. i. (RI: 1103)	0.17±0.04	0.05±0.06	3.3	<0.001
Glutamic acid	0.16±0.03	0.09±0.05	2	<0.001
Asparagine	0.15±0.1	0.01±0.01	30.2	0.003
n. i. (3-Methyl-3-Hydroxy -Pentandioic acid R-Match: 930, RI: 1624)	0.12±0.08	0.01±0.02	9.6	0.005
n. i. (Aspartic acid, R-Match: 859, RI: 1536)	0.1±0.05	0.03±0.03	3.9	0.002
n. i. (3-Cyanoalanine, R-Match: 879, RI: 1348)	0.08±0.05	0±0.01	22.9	0.001
n. i. (Asparagine [−H2O] (2TMS), R-Match: 879, RI: 1507)	0.06±0.05	0.01±0.01	7.9	0.007
n. i. (Galactonic acid, R-Match: 901, RI: 2042)	0.06±0.01	0.01±0.01	5.8	<0.001
Valine	0.05±0.03	0.01±0.01	4.3	0.001
n. i. (Urea, R-Match: 904, RI: 1243)	0.05±0.03	<0.01	700	0.001
n. i. (Methylenbutanedioic acid, R-Match: 943, RI: 1353)	0.05±0.01	0.01±0.01	10.3	<0.001
n. i. (Arabinofuranose, R-Match 834, RI 1799)	0.05±0.02	0.01±0.01	3.8	0.001
n. i. (RI: 1552)	0.04±0.02	0.01±0	7.2	0.001
n. i. (RI: 1354)	0.04±0.02	0.01±0.01	4.4	0.007
n. i. (Glyceric acid, R-Match: 840, RI: 1347)	0.03±0.02	0.01±0.01	2.4	0.016
n. i. (RI: 1086)	0.03±0.02	<0.01	392.9	0.001
Leucine	0.02±0.01	<0.01	5.6	0.001
n. i. (RI: 1693)	0.02±0.01	<0.01	45.3	0.001
n. i. (RI: 1791)	0.02±0.01	<0.01	4.5	<0.001
n. i. (2-Butenedioic acid, R-Match: 854, RI:1313)	0.02±0	0.01±0.01	2.1	0.007
n. i. (RI:1345)	0.01±0	<0.01	4.1	0.001
n. i. (Aconitic acid, R-Match: 903, RI: 1770)	0.01±0.01	<0.01	136.3	0.001
n. i. (RI: 1787)	0.01±0	<0.01	10000	<0.001
n. i. (2-Piperidinecarboxylic acid, R-Match: 974, RI: 1348)	0.01±0.01	<0.01	3	0.014
n. i. (RI: 1131)	0.01±0.01	<0.01	17.5	0.005
Phenylalanine	<0.01	<0.01	4.5	0.001
Cysteine	<0.01	<0.01	10.4	<0.001

Polar metabolites were extracted with methanol, derivatized and subjected to GC-MS analysis. Listed are compounds that showed a significant (P<0.05, Student’s t-test) difference (>1.5-fold) in three independent biological experiments. Entries are ordered according to their maximum relative abundance under alkaline conditions; n. i.: not unambiguously identified (no comparison with authentic standard), RI: retention index, R-Match: reverse match (only values above 800 are given).

The overlap in responses contained three additional metabolites (Fig. 2A). Amino acids asparagine and threonine were found to be up-regulated with a 30-fold higher abundance of asparagine under alkaline conditions representing the most prominent change. Overall, the signal intensities were far less pronounced than those for the organic acids (Tables 1 and 2). A tentatively identified metabolite showing robust differences in abundance was methyl-3-hydroxy-pentandioic acid (more abundant under Fe deficiency).

Importantly, because the up-regulation of citrate and malate synthesis is a well-established root metabolic marker for Fe depletion [42], [43], the GC-MS profiling results confirmed the Fe deficiency indicated by the ICP-OES measurements (Fig. 1) and further validated our assay conditions. Growth in Fe-free medium at pH 5.7 as well as growth in hydroponic medium at pH 7.7 imposed Fe deficiency and elicited a respective root response.

### UPLC-ESI-QTOF-MS profiling

Most of our analyses focused on LC-MS-based profiling which covers other compound classes than GC-MS and has the potential to provide structural information on unknown compounds [36], [44]. Methanolic extracts of roots were analyzed in negative and positive ionization mode. For both sets of three independent experiments hundreds of significant (P<0.05) changes associated with Fe deficiency were detected. The total numbers of features with >2-fold changes were 162 and 167 for Fe-free medium in positive and negative mode, respectively, and 678 and 323 for alkaline medium. In order to identify the metabolic changes most closely associated with Fe deficiency we filtered out all features that showed significant >2-fold changes in signal intensity under both tested conditions. Fifty-three and 36 signals met these criteria in positive and negative mode, respectively (Fig. 2B). The corresponding lists are displayed in Tables S1 and S2 in File S6.

**Figure 4:**
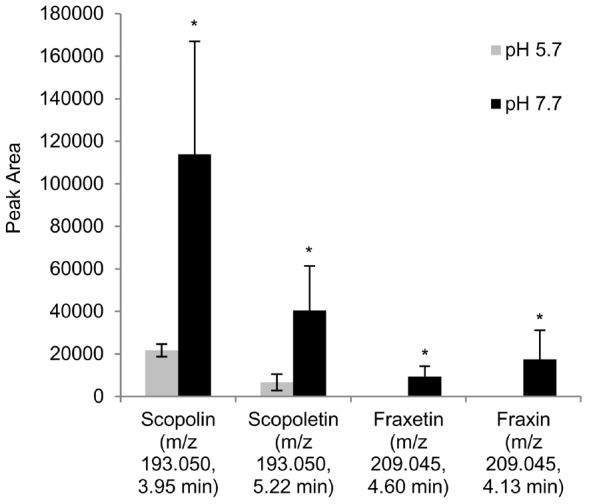
Coumarins were among the compounds most strongly responding to Fe deficiency as identified in UPLC-ESI-QTOF-MS-generated metabolite profiles of *A. thaliana* wild-type roots. The coumarin response to Fe deficiency is illustrated here by showing peak areas of the quantifier ions (ESI+ ionization mode, see Table S1 in File S6) in metabolite profiles of *A. thaliana* Col-0 plants cultivated either at pH 5.7 (light grey bars) or at pH 7.7 (black bars). Identification was based on a comparison with authentic standards. The means of three independent biological experiments are displayed. Error bars indicate standard deviation. Significant differences between control (pH 5.7) and Fe deficiency (pH 7.7) conditions were determined by Student’s t-test, *P<0.05.

**Figure 5:**
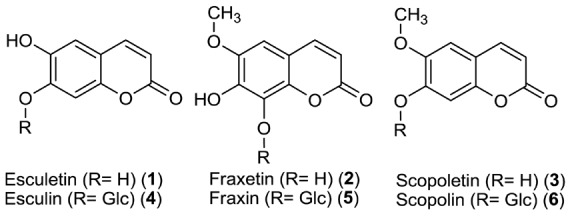
Structures of coumarins and their respective glycosides investigated in this study.

In order to annotate the selected features, MS-MS data were obtained for approximately two thirds of them. Recorded MS-MS-spectra (provided as MS2Tags in a format compatible with Massbank in Files S2 and S3) were compared with Massbank [38] and the Riken MS2T database [36]. We were able to assign several peaks to known MS2Ts. However, in most cases, there was no structural data for the respective compound available yet. Only for a limited number of features, MS2T and Massbank hits led to a reasonable structure prediction. Whenever possible, we then obtained the respective compounds as standards and compared retention times and MS-MS spectra. Following this strategy we were able to identify the main coumarin known in *Arabidopsis thaliana*, scopoletin, and its respective glycoside scopolin as two molecules showing major changes in abundance under Fe-deplete conditions (Fig. 4). The scopoletin standard we had to synthesize. The recrystallized product was checked by ESI-MS. A mass of 193.0522 was found (calculated for C53H76NO7 [M+H]^+^193.0501). All physical and spectroscopic data of the product were in accordance with the literature [40], [41]. Another highly hydroxylated coumarin, fraxetin, and its corresponding glycoside fraxin could be identified as significantly up-regulated too (Fig. 4). The chemical structures of the described coumarins are shown in Fig. 5 (**2**, **3**, **5**, **6**). An unidentifed compound co-eluted with scopolin. The detected marker 225.040 ESI+ at 3.95 min most likely resulted from an in-source fragmentation of the respective hexoside with a molecular weight of 386.088 (experimental) and an elemental composition of C16H18O11. The MSMS could be matched to the MS2T ATH64p07367 and, based on the most probable elemental composition, fraxin substituted with an additional OH-group is a likely candidate. A group of markers at 5.14 min was assigned to a compound with a molecular weight of 522.210. Three MS2Ts could be matched to the compound, and based on the CHNOPS elemental composition we assigned a molecular formula (C26H34O11) to the compound.

### A coumarin biosynthetic mutant under Fe-limited alkaline conditions

**Figure 6:**
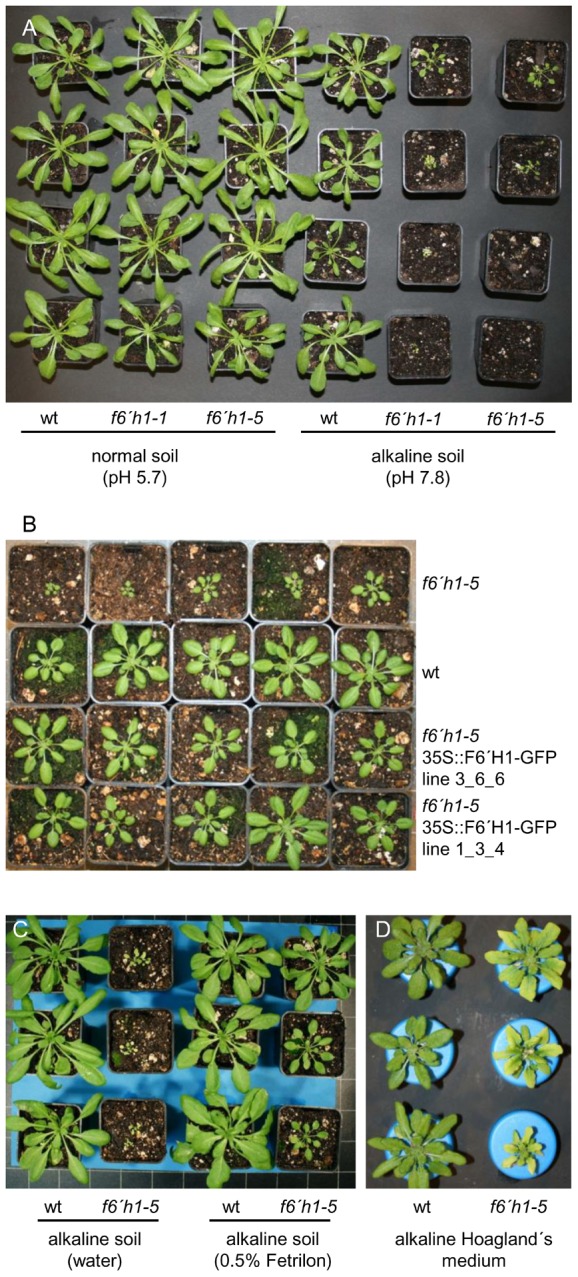
Growth of coumarin-deficient *f6*′*h1* T-DNA insertion mutants is strongly impaired under Fe-limited alkaline conditions. (A) *A. thaliana* wild type plants (wt) and *f6*′*h1* T-DNA insertion mutants *f6*′*h1-1* and *f6*′*h1-5* were grown on normal soil (left) and on soil alkalinized through the addition of CaO (right). (B) Overexpression of *F6*′*H1* under control of the 35S promoter in *f6*′*h1-5* fully rescues the mutant phenotype on alkaline soil. Shown are two independent transgenic lines grown alongside the wild type and the *f6*′*h1-5* mutant on alkaline soil. (C) The mutant phenotype on alkaline soil can be partially rescued by Fe fertilization through watering with 0.5% Fetrilon (Fe-EDTA solution). (D) Col-0 wild type plants (wt, left) and *f6*′*h1-5* mutant plants (right) were cultivated for six weeks in hydroponic medium with a pH of 7.7 instead of 5.7. All experiments were repeated three times independently with nearly identical outcome. The individual plants shown represent the range of phenotypes observed.

Biosynthesis of scopoletin has been molecularly elucidated [45] and the enzyme catalyzing a key step, the *ortho*-hydroxylation of cinnamates, is known as F6′H1 [23]. This enabled us to directly test a possible role of coumarins in Fe acquisition. We obtained two mutant alleles of the respective *F6*′*H1* gene (At3g13610) in Col-0 background, *f6*′*h1-1* [23] and *f6*′*h1–5*. For the latter the T-DNA insertion in exon 2 was confirmed (File S1). When grown on normal soil *f6*′*h1* mutants showed no obvious phenotypes. On alkaline soil, however, a dramatic effect on development was observed (Fig. 6A). While Col-0 plants showed only a slight impairment of growth due to limited Fe availability, *f6*′*h1* mutant plants were chlorotic and barely grew. Most plants did not survive six weeks of cultivation under these conditions. Expression of *F6*′*H1* under control of the 35S promoter fully restored wild–type growth in *f6*′*h1-5* mutants as shown in Fig. 6B for two independent transgenic lines. This established a lack of functional F6′H1 as the cause of the growth defect on alkaline soil. Since the two mutant alleles behaved indistinguishable we performed further experiments with only one of them (*f6*′*h1–5*).

The alkaline soil phenotype is reminiscent of previously reported ones for mutants with defects in Fe acquisition [46]. Correspondingly, Fe fertilization of alkaline soil, i.e. watering with Fe-EDTA solution (0.5% Fetrilon), attenuated the symptoms of *f6*′*h1* plants (Fig. 6C). This clearly demonstrated Fe deficiency as the cause of the strong growth inhibition displayed by plants lacking coumarin biosynthesis.

In hydroponic culture at alkaline pH, *f6*′*h1-5* mutants also showed impaired growth relative to wild-type plants and displayed severe chlorosis, thus again indicating Fe deficiency (Fig. 6D, File S4). No growth phenotype was apparent when Col-0 and *f6*′*h1-5* were grown hydroponically in Fe-free medium at pH 5.7 (File S4), indicating that coumarin biosynthesis is required for Fe acquisition predominantly under Fe-limited conditions. When grown under the same conditions as Col-0 plants for the metabolome analysis, *f6*′*h1-5* plants showed shoot and root Fe concentrations that were slightly higher than those measured for wild-type plants. Specifically under conditions of limited Fe availability due to alkaline pH, however, reductions in shoot Fe concentrations were even stronger than in Col-0 (File S4, compare to Fig. 1B). Root Fe concentrations of *f6*′*h1-5* plants grown under these conditions were higher than those of Col-0 plants, possibly indicating larger apoplastic Fe pools.

**Figure 7:**
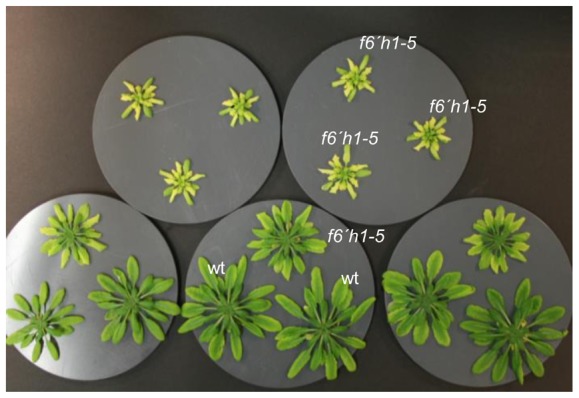
Partial rescue of the *f6*′*h1-5* growth defect in alkaline medium by co-cultivation with wild-type plants. *F6*′*H1* knock-out plants (*f6*′*h1–5*) were co-cultivated for four weeks in alkaline Hoagland’s solution (pH 7.7) either with plants of the same mutant genotype (top row) or with *A. thaliana* Col-0 plants (wt) (bottom row). Experiments were repeated three times independently with nearly identical outcome.

Together these observations strongly suggested an important role of coumarin biosynthesis for Fe acquisition under alkaline conditions. In order to test whether coumarins exert their effects extracellularly or intracellularly we performed hydroponic co-cultivation experiments. One *f6*′*h1-5* mutant plant was grown together with either two other mutant plants or with two Col-0 plants in 1.6 l containers with alkaline medium. The pH was monitored throughout and did not change over the course of the experiment. Interestingly, *f6*′*h1-5* plants co-cultivated with Col-0 plants grew much bigger and were less chlorotic when compared to mutant plants grown without Col plants (Fig. 7). This clearly indicated at least a partial contribution of secreted coumarins to Fe acquisition and prompted us to analyze root exudation.

### Metabolite profiling of root exudates

Exudates of wild type and *f6*′*h1-5* mutant plants grown hydroponically under control conditions (pH 5.7) – in order to achieve equal growth rates of the two genotypes - were collected, concentrated and analyzed by UPLC-ESI-QTOF-MS. The resulting metabolite profiles were compared and significant differences determined to identify compounds that are secreted by Col-0 and not by plants with a defect in coumarin biosynthesis. Results are listed in Tables S3 and S4 in File S7. Scopoletin was identified as one of the most intense signals in the exudates of wild-type plants even under control conditions. Only traces were detectable in the exudates of the mutant, confirming the lack of synthesis in *f6*′*h1-5* mutant plants [23]. As an additional metabolite downstream of F6′H1 we detected esculetin (Fig. 5, **1**) in the exudates. Thus, coumarins appear to be secreted by *A. thaliana* roots and could indeed be involved in the mobilization of extracellular insoluble Fe(III).

### Fe(II)/Fe(III) complexing and Fe(III) reducing capacity of candidate coumarins

**Figure 8:**
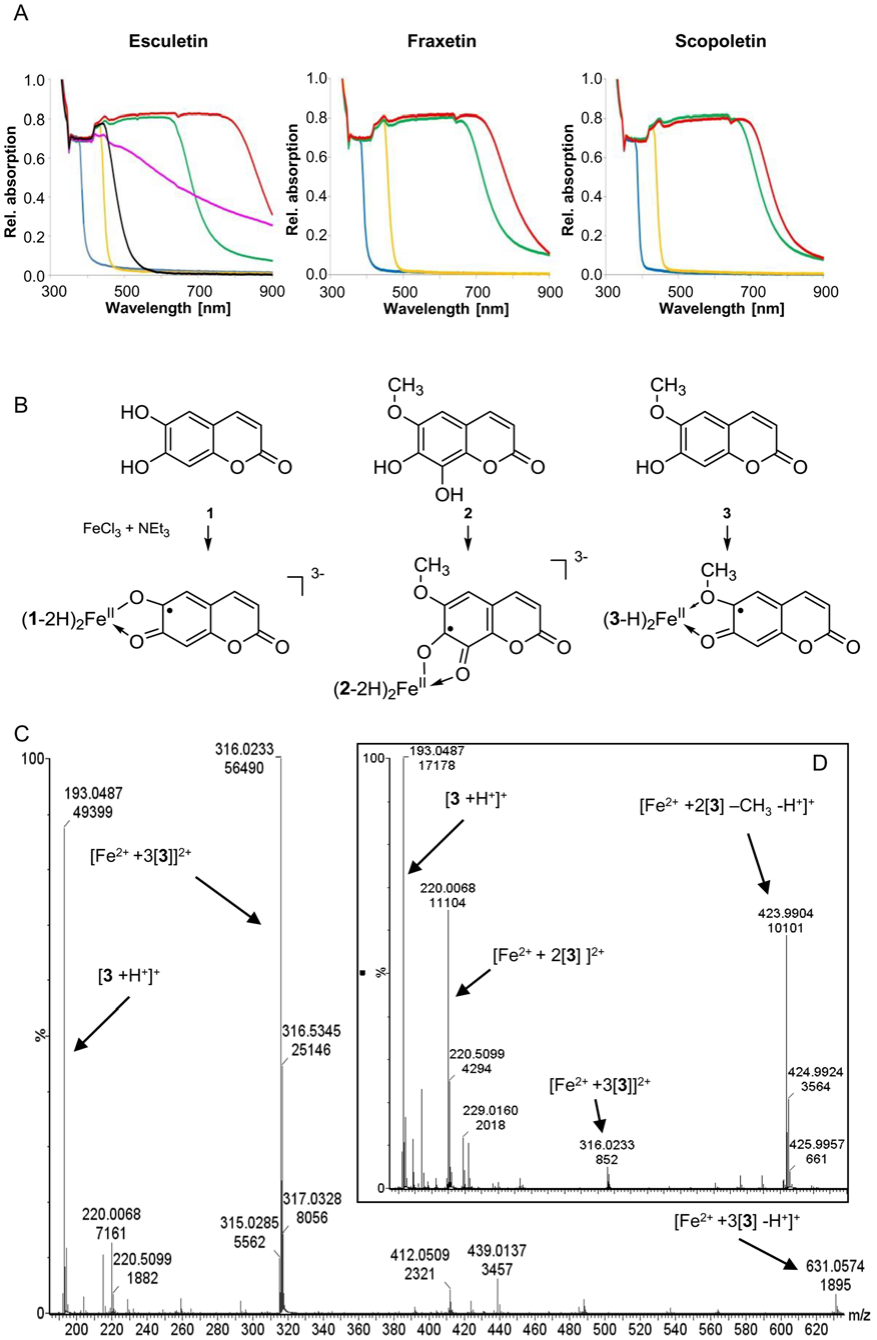
UV/vis spectroscopy and direct-infusion ESI-QTOF-MS demonstrate reduction of Fe(III) by coumarins and the formation of Fe(II)-coumarin complexes *in vitro*. (A) Optical spectra of solutions (CH_3_CN) of ligands esculetin (**1**), fraxetin (**2**) and scopoletin (**3**), of iron salts, of mixtures of ligands and 2 equiv of NEt_3_, and of mixtures containing 3 equiv of ligand, 6 equiv of NEt_3_, and 1 equiv of FeCl_2_ or FeCl_3_. Left: **1** (blue); **1**+ NEt_3_ (orange); **1**+ NEt_3_+ FeCl_2_ (green); **1**+ NEt_3_+ FeCl_3_ (red); FeCl_3_ (black); FeCl_2_ (magenta). Center: **2** (blue); **2**+ NEt_3_ (orange); **2**+ NEt_3_+ FeCl_2_ (green); **2**+ NEt_3_+ FeCl_3_ (red). Right: **3** (blue); **3**+ NEt_3_ (orange); **3**+ NEt_3_+ FeCl_2_ (green); **3**+ NEt_3_+ FeCl_3_ (red). (B) Interpretation of the UV/vis spectra depicted in (A). (C) Results of direct-infusion ESI-QTOF-MS of a mixture of Fe(II) with synthesized and purified scopoletin. (D) MS-MS spectrum of the main signal at *m/z* 316.02.

Fe reducing as well as Fe chelating activities have been ascribed to phenolic compounds [14], [17]. In order to further investigate the mechanism underlying the Fe solubilizing effect of scopoletin and possibly other secreted coumarins we attempted to identify the Fe complexes that most likely form under basic conditions and in the presence of the ligands esculetin (**1**), fraxetin (**2**), and scopoletin (**3**) on the basis of their optical spectra. Fig. 8A depicts these spectra as well as control spectra of solutions of the ligands, of the ligands plus NEt_3_, and of the Fe salts. In line with the literature [47] the absorption maxima of the free ligands (ca. 370 nm) were red-shifted by ca. 50–80 nm upon addition of the base and formation of the catecholate dianions of esculetin and fraxetin, or of the phenolate monoanion in the case of scopoletin. The maxima in both the neutral ligands and the catecholate/phenolate anions originate from ligand π→π* transitions.

Solutions of mixtures of the ligands esculetin (**1**), fraxetin (**2**) and scopoletin (**3**), base NEt_3_ and FeCl_2_ gave rise to UV/vis curves with long, almost horizontal plateaus with maxima around 630–670 nm and tailing off until ca. 800 nm, originating from ligand-to-metal charge-transfer bands [47], [48] which are typical of Fe-triscatecholate complexes.

Interestingly, the optical spectra of solutions containing the ligands **1–3**, base NEt_3_ and FeCl_3_ were of similar shape, yet with maxima and tails distinctly shifted to greater wavelengths when compared with the Fe(II) systems, e.g. shifted by ca. 120 nm in the case of esculetin, by ca. 60 nm for fraxetin, and by ca. 30 nm in the case of scopoletin (Fig. 8A). Fig. 8B depicts the interpretation of the spectra shown in Fig. 8A.

To independently assess interaction of the coumarins with Fe and to determine more accurately the stoichiometry, the ligands dissolved in methanol were added to aqueous solutions of ferric or ferrous iron. These mixtures were analyzed by ESI-QTOF-MS in positive and negative ionization mode via direct infusion. The free ligand was visible in all spectra recorded. Astonishingly, only in the combination of Fe(II) and scopoletin the formation of new spectral peaks could be observed independent of the stoichiometric ratio and the methanol concentration (Fig. 8C). For the main peak at *m/z* 316.02 a scopoletin: Fe ratio of 3∶1 was calculated. The isotopic pattern of Fe matched well with the respective computed model (File S5). MS-MS experiments confirmed the presence of the respective ligands and Fe in this adduct (Fig. 8D).

## Discussion

Our metabolome analysis aimed at elucidating the substantial changes in various metabolic pathways that occur upon Fe deficiency [49]–[51]. For instance, root secretion of phenols has long been hypothesized to be a biochemical part of the Fe acquisition strategy in higher plants other than grasses [52]. Phenols belong to the main constituents of root exudates and numerous studies have documented an increase in their synthesis under conditions of limited Fe availability (reviewed in [14]). This response has been implicated in the mobilization of Fe bound in the apoplast. However, the mechanisms underlying the re-utilization of Fe are unknown [53], and direct evidence for the biological importance of this process was scarce until recent work demonstrated a role of coumarin biosynthesis and secretion for Fe acquisition in *A. thaliana* [19]–[21].

We combined GC-MS- and UPLC-ESI-QTOF-MS-based metabolite profiling to cover molecules of different polarity. Our analyses detected a large number of metabolic alterations triggered by Fe depletion in *A. thaliana* roots. This is in agreement with the pronounced complexity of physiological plant adaptations to low Fe availability [50]. Monitoring responses using two different conditions helped tremendously in ascertaining the molecules that showed the most robust changes in association with Fe deficiency.

Among sugars, amino acids and organic acids, the compound classes usually covered when analyzing methanolic extracts by GC-MS, strong increases in citrate and malate signals were by far the dominant responses. This confirmed a large body of work on various strategy I plants [42] and at the same time provided strong evidence that our experimental conditions indeed triggered an Fe deficiency response. Citrate is one of the main Fe chelators in plants. It forms complexes with Fe(III) in the xylem. Therefore, sufficient loading of citrate into the xylem is essential for Fe translocation [54]–[56]. Increases observed in the abundance of a few amino acids on the other hand are in contrast to published reports that found rather a decrease in the abundance of the majority of detected amino acids in *Cucumis sativus* roots exposed to Fe deficiency [57]. On the other hand, Fe deficiency appears to activate N cycling and protein catabolism in *Medicago truncatula* roots [58]. In the absence of comparative kinetic analyses of metabolome changes under Fe deficiency in different plant species we cannot explain these differences. Other changes identified by our GC-MS analyses lack obvious connections to Fe homeostasis but can become informative in future studies.

Metabolome analysis via UPLC coupled to ESI-QTOF-MS has great potential to unravel the dynamics of plant secondary metabolism because wide coverage of relevant compound classes as well as high resolution can be achieved [59]. Among a large number of significant changes in the metabolome of plants exposed to Fe-deplete conditions, the increase in coumarin levels was particularly striking. Peaks representing scopolin and fraxin as well as their respective agylcones scopoletin and fraxetin were among those showing the highest fold changes detected (Fig. 4; Tables S1 and S2 in File S6). This is in agreement with the previously reported analysis of UV-fluorescent metabolites accumulating in *A. thaliana* roots under Fe-deplete conditions [20].

These coumarins could be unequivocally identified owing to the availability of reference spectra and authentic standards. It remains a challenge, however, to annotate peaks detected in LC-MS-derived metabolite profiles [38]. Thus, most of the detected features remain unidentified. Still, with the help of rapidly growing databases the results listed in Tables S1 and S2 in File S6 are expected to represent a valuable resource for future system level analyses of root metabolome dynamics under Fe deficiency conditions, especially in combination with MS-MS data provided in Files S2 and S3.

Strong up-regulation of coumarin biosynthesis as indicated by our data is consistent with several studies that analyzed global responses at the transcriptome, proteome and metabolome level. Microarray experiments showed transcriptional up-regulation of the phenylpropanoid pathway [50]. Following a proteomics approach Lan et al. reported higher levels of enzymes in this pathway under Fe deficiency. The list of identified proteins included F6′H1 [60]. Furthermore, our observations are similar to recently reported findings. Fourcray et al. identified several coumarins including scopoletin and fraxetin as being secreted in a ABCG37-dependent fashion by *A. thaliana* roots [20]. Schmid et al. showed accumulation of scopolin and scopoletin in root extracts of Fe-deficient roots [21]. Unlike the results reported here and by Fourcray et al., however, Schmid et al. also detected esculin and esculetin in root extracts.

Growth experiments with Col-0 wild type, *f6*′*h1* mutants and complemented lines clearly demonstrated an essential role of molecules downstream of the *ortho*-hydroxylation of cinnamates catalyzed by F6′H1 [23] for Fe acquisition both when cultivated on soil and in hydroponics (Figs. 6 and 7). Under alkaline conditions lack of this enzyme resulted in severe Fe deficiency as indicated by pronounced leaf chlorosis and strongly reduced shoot Fe levels (File S4). This again is in agreement with recently published observations for *f6*′*h1* mutant plants exposed to Fe deficiency at the seedling stage *in vitro* [19] or on soil [21] as well as mutant seedlings defective in the presumed coumarin export system ABCG37 [20]. Thus, we further support the conclusion that coumarin biosynthesis represents an integral part of strategy I. It is crucial under alkaline conditions, which represent a major limiting factor for agricultural productivity worldwide. Furthermore, it is interesting to note that the specific adaptations of calcicole plants to calcareous substrates have been hypothesized to be at least in part dependent on a pronounced shift from classic strategy I responses towards phenolics secretion specifically under alkaline conditions [61]. This was observed for *Parietaria diffusa*, an Fe-efficient plant that can be found on walls and similar substrates in the Mediterranean, when effects elicited by Fe-free medium were compared to those of an alkaline carbonate medium.

The partial rescue we observed upon co-cultivation with wild-type plants was reported in a study published while this manuscript was in preparation [19], albeit in a different experimental setup, i.e. with younger plants. Co-cultivation effects suggested that a lack of secreted coumarins accounts for the *f6*′*h1* phenotype (Fig. 7). Comparative analysis of root exudates derived from Col-0 and *f6*′*h1* plants showed indeed unequivocally and in agreement with Schmid et al. [21] that the aglycones scopoletin and esculetin are released by *A. thaliana* wild type roots, but not by *f6*′*h1* roots. The identification of esculetin was surprising as neither esculetin nor its hexoside esculin could be found in root extracts. Possibly, scopoletin can be extracellulary demethylated to esculetin. Alternatively, our analysis and the one performed by Fourcray et al. [20] may have missed the comparatively small signals (relative to scopoletin) for esculetin and esculin reported by Schmid et al. [21]. A large number of other F6′H1 dioxygenase-dependent features were detected in Col-0 root exudates. Their identities could not be determined. However, the data set reported here (Tables S3 and S4 in File S7) can be helpful in further dissecting the coumarin pathway in a fashion similar to, for instance, the elucidation of reactions upstream and downstream of chalcone synthase [31]. This will aid in meeting the challenge of identifying other coumarins potentially involved in Fe acquisition [21].

Given the essential role of secreted scopoletin one has to postulate the existence of efflux transporters similar to rice PEZ1 [2], [16]. The ABC transporter ABCG37 has been proposed as a candidate because of its strong expression in Fe-deficient roots [50], [51] and lack of functional ABCG37 indeed compromises growth of *A. thaliana* under alkaline conditions [19]. Indeed, the recently reported growth impairment of *pdr9–2* seedlings in the presence of pH conditions limiting Fe availability associated with a lack of coumarin secretion strongly supports this hypothesis [20]. Phenols of the catechol type exudated into the rhizosphere are considered to mobilize ferric iron by complexation and/or reduction. Indeed, Fe chelating activity of coumarins at neutral pH was already demonstrated [62]. We attempted to further characterize the mechanism underlying the demonstrated beneficial effect of coumarins through *in vitro* studies. For that we used a non-aqueous system to avoid the formation of oxo-, hydroxyl- and aquo-Fe complexes. While the conditions applied were with the necessary use of organic solvents non-physiological, they still led us to tentatively propose that scopoletin, esculetin and fraxetin can solubilize apoplastic Fe(III) because they can act both as reductants of Fe(III) and as ligands for Fe(II) under basic conditions.

There was little difference between the UV/vis spectra of mixtures of base NEt_3_ and FeCl_2_ containing the catechols esculetin and fraxetin, and those containing the *o-*hydroxyanisole scopoletin (Fig. 8A). Careful studies with heteroleptic Fe complexes bearing monodentate phenolate or *o*-chlorophenolate ligands versus optionally bisdentate *o*-hydroxyanisole ligands showed that Fe complexes with monodentate phenolate ligands have UV maxima at distinctly shorter wavelengths around 430–450 nm [63]. Hence, we conclude that scopoletin in [(scopoletin-H)_n_Fe(II)]^m–^ complexes also acts as a bisdentate ligand with the methoxy oxygen providing a dative bond to Fe.

A conceivable explanation for the UV/vis curves of our Fe(III)–ligand systems in acetonitrile could lie in the oxidation of the catecholates of ligands esculetin and fraxetin by Fe(III) to give the respective *o*-semiquinonates that coordinate to the concomitantly formed Fe(II). Jones et al. [47] recorded UV/vis spectra of water-free solutions (Me_2_SO or MeCN) containing Fe(II) or Fe(III) plus electrochemically generated 3,5-di-tert-butylchatecholate dianions, or *o*-semiquinonate anions in various stoichiometric ratios. They found that solutions containing Fe(III) and catecholate dianions in a 1∶3 ratio yield the complex species [Fe^III^(catecholate)_3_]^3–^ showing a band with a sharp maximum at ca. 490 nm and steeply tailing off. Curves with very long plateaus reaching to wavelengths of ca. 800–900 nm and so greater than those of the Fe(II)-catecholate complexes were observed by them for complexes with *o*-semiquinonate anions. The mixed complexes of type [Fe^II^(*o*-semiquinonate)(catecholate)_1–2_]^1–^ °^r^ ^3–^ should be stable and show UV/vis bands as observed (Fig. 8A). It is plausible that scopoletin gives rise to a similar complex (Fig. 8B). The relatively small red-shift of only 30 nm when compared with the [(scopoletin-H)_3_Fe(II)]^–^ analog can be rationalized by a less extensive change of the ligand than in the case of esculetin and fraxetin.

While it is difficult to study complexes of Fe with phenols under alkaline conditions by ESI-MS [64], we did observe that scopoletin in contrast to esculetin readily forms stable adducts with Fe(II) ions. Such interactions might prevent Fe from re-oxidation and keep it available for uptake into the symplast under physiological conditions. However, we cannot at this stage explain the failure to detect adducts with esculetin and fraxetin.

The Fe(III) mobilizing activity found by Schmid et al. [21] for esculetin but not scopoletin suggests a chelating activity of this catechol-type molecule with neighbouring hydroxyl groups as being responsible for the beneficial effect of coumarin secretion under alkaline conditions. On the other hand, scopoletin and esculin were as efficacious as esculetin in rescuing the growth defect of *f6*′*h1* mutant seedlings [21]. Thus, more detailed studies and a comprehensive picture of root exudation upon Fe deficiency as well as possible conversions of secreted coumarins will be needed to unravel the exact mechanism.

In conclusion, we lend further support to the recently established crucial role of coumarin secretion for Fe acquisition by the strategy I plant *A. thaliana* under alkaline conditions. We show that coumarin biosynthesis in roots is enhanced under conditions of Fe deficiency. Coumarins are found in root exudates and appear to contribute to Fe acquisition extracellulary. Our *in vitro* evidence suggests a Fe(III) reducing and Fe(II) complexing activity of coumarins as the underlying mechanism. The metabolome data represent a potentially valuable resource for future system level analyses of Fe deficiency responses.

## Supporting Information

File S1 **Genotyping of T-DNA insertion line SALK_050137 ( =** ***f6*** **′** ***h1-5*** **).**
(PDF)

File S2 **MS2Tags_positive mode_Massbank.**
(TXT)

File S3 **MS2Tags_negative mode_Massbank.**
(TXT)

File S4 ***A. thaliana f6***′***h1-5*** **plants were exposed to two different conditions causing Fe deficiency and used for the metabolite profiling experiments with Col-0 plants.** (A) Plants were grown hydroponically in 1/10 Hoagland Solution for six weeks. Plants were cultivated either at a pH of 5.7 with Fe-HBED as Fe source (Control), at a pH of 7.7 with Fe-HBED as Fe source (pH 7.7), or the final two weeks at a pH of 5.7 without Fe-HBED (−Fe). (B) Fe concentrations in roots (blue bars) and shoots (red bars) were determined by ICP-OES. The means of three independent biological experiments are displayed. Error bars indicate standard deviation. Significant differences to plants grown under control conditions were determined by Student’s t-test, *P<0.05.
(PDF)

File S5 **Isotopic pattern of iron for the main peak at** ***m/z*** **316.02 shown in** **Figure 8C** **.** A scopoletin: Fe ratio of 3∶1 was calculated.
(PDF)

File S6 **Tables S1 and S2 listing markers consistently up-regulated upon hydroponic cultivation in Fe-free and alkaline medium condition in** ***A. thaliana*** **as detected by UPLC-ESI-QTOF-MS in either positive ionization mode (Table S1) or negative ionization mode (Table S2).** Markers from three biological replicates were chosen when they met the following criteria in both sets of three biologically independent experiments each: Fold-change >2 and P<0.05 (Student’s t-test). Annotation level (Ann. Level): 1. compound identified using a synthesized standard; 2. compound putatively annotated by interpretation of mass spectrometry data; 3. compound class putatively annotated; 4. unknown compounds. *m/z*: Mass to charge ratio. Annotation: MS2T identifiers, elemental composition of the uncharged compound, adducts or the identified compounds are given. Molecular Mass: Molecular mass of the respective compound (i.e. precursor or M) is given.
(DOCX)

File S7 **Tables S3 and S4 listing markers identified in a comparison of root exudates harvested from** ***A. thaliana*** **wild type and** ***f6***′***h1-5*** **mutant plants as detected by UPLC-ESI-QTOF-MS in either positive ionization mode (Table S3) or negative ionization mode (Table S4).** Markers accumulating in the wild-type exudates were chosen when they met the following criteria: Fold-change >2 and P<0.05 (Student’s t-test; 3 independent biological experiments). Annotation level (Ann. Level): 1. compound identified using a synthesized standard; 2. compound putatively annotated by interpretation of mass spectrometry data; 3. compound class putatively annotated; 4. unknown compounds. *m/z*: Mass to charge ratio. Annotation: adducts or the identified compounds are given. Molecular Mass: Molecular mass of the respective compound (i.e. precursor or M) is given. Entries are ordered according to their fold-change.
(DOCX)
