# Supplementary figures and images for: Metabolome Analysis of Arabidopsis thaliana Roots Identifies a Key Metabolic Pathway for Iron Acquisition
Source: PLoS One. 2014 Jul 24;9(7):e102444. doi: 10.1371/journal.pone.0102444 (PMC4109925; doi:10.1371/journal.pone.0102444)

## Supplemental File S1

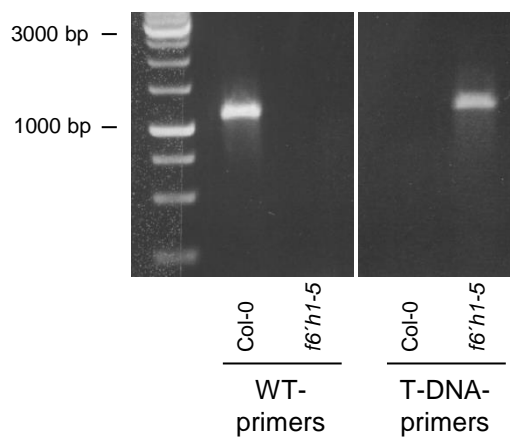

Genotyping of T-DNA insertion line SALK\_050137 (= *f6'h1-5*)

Supplement: File S1 — Genotyping of T-DNA insertion line SALK_050137 ( = f6 ′ h1-5 ). (PDF) [file pone.0102444.s001.pdf]
