# Supplementary material for: Metabolome Analysis of Arabidopsis thaliana Roots Identifies a Key Metabolic Pathway for Iron Acquisition
Source: PLoS One. 2014 Jul 24;9(7):e102444. doi: 10.1371/journal.pone.0102444 (PMC4109925; doi:10.1371/journal.pone.0102444)
Supplement: File S4 — A. thaliana f6′h1-5 plants were exposed to two different conditions causing Fe deficiency and used for the metabolite profiling experiments with Col-0 plants. (A) Plants were grown hydroponically in 1/10 Hoagland Solution for six weeks. Plants were cultivated either at a pH of 5.7 with Fe-HBED as Fe source (Control), at a pH of 7.7 with Fe-HBED as Fe source (pH 7.7), or the final two weeks at a pH of 5.7 without Fe-HBED (−Fe). (B) Fe concentrations in roots (blue bars) and shoots (red bars) were determined by ICP-OES. The means of three independent biological experiments are displayed. Error bars indicate standard deviation. Significant differences to plants grown under control conditions were determined by Student’s t-test, *P<0.05. (PDF) [file pone.0102444.s004.pdf]

Supplemental File S4

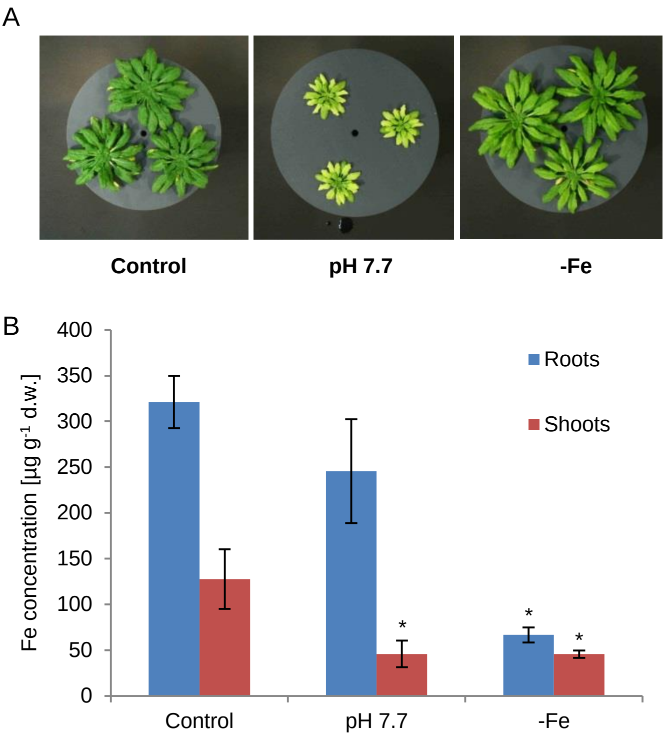

***A. thaliana f6´h1-5* plants were exposed to two different conditions causing Fe deficiency and used for the metabolite profiling experiments with Col-0 plants.** (A) Plants were grown hydroponically in 1/10 Hoagland Solution for six weeks. Plants were cultivated either at a pH of 5.7 with Fe-HBED as Fe source (Control), at a pH of 7.7 with Fe-HBED as Fe source (pH 7.7), or the final two weeks at a pH of 5.7 without Fe-HBED (-Fe). (B) Fe concentrations in roots (blue bars) and shoots (red bars) were determined by ICP-OES. The means of three independent biological experiments are displayed. Error bars indicate standard deviation. Significant differences to plants grown under control conditions were determined by Student´s t-test, *P<0.05.
