# Supplementary material for: Metabolome Analysis of Arabidopsis thaliana Roots Identifies a Key Metabolic Pathway for Iron Acquisition
Source: PLoS One. 2014 Jul 24;9(7):e102444. doi: 10.1371/journal.pone.0102444 (PMC4109925; doi:10.1371/journal.pone.0102444)
Supplement: File S5 — Isotopic pattern of iron for the main peak at m/z 316.02 shown in Figure 8C . A scopoletin: Fe ratio of 3∶1 was calculated. (PDF) [file pone.0102444.s005.pdf]

Supplemental File S5

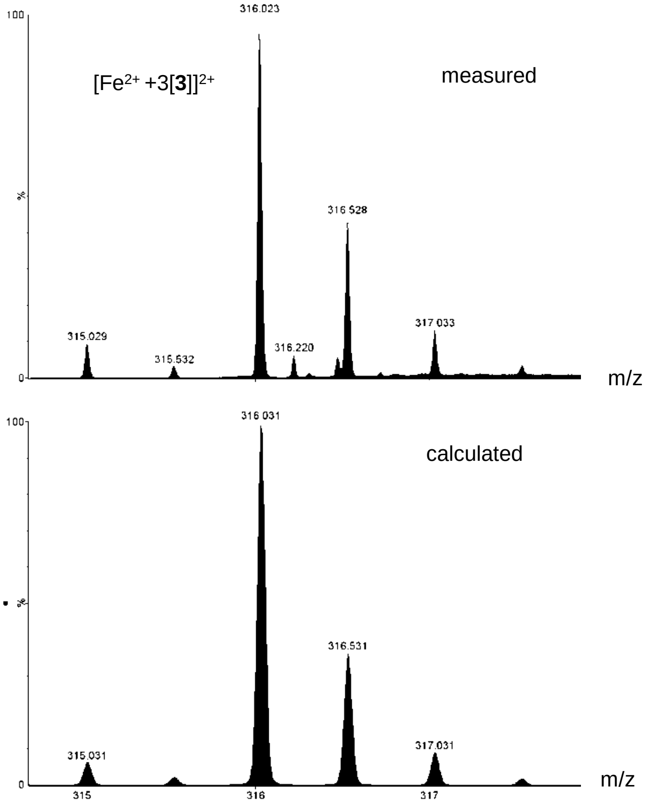

Isotopic pattern of iron for the main peak at *m/z* 316.02 shown in Figure 8C. A scopoletin:Fe ratio of 3:1 was calculated.
