# Supplementary material for: Metabolome Analysis of Arabidopsis thaliana Roots Identifies a Key Metabolic Pathway for Iron Acquisition
Source: PLoS One. 2014 Jul 24;9(7):e102444. doi: 10.1371/journal.pone.0102444 (PMC4109925; doi:10.1371/journal.pone.0102444)
Supplement: File S6 — Tables S1 and S2 listing markers consistently up-regulated upon hydroponic cultivation in Fe-free and alkaline medium condition in A. thaliana as detected by UPLC-ESI-QTOF-MS in either positive ionization mode (Table S1) or negative ionization mode (Table S2). Markers from three biological replicates were chosen when they met the following criteria in both sets of three biologically independent experiments each: Fold-change >2 and P<0.05 (Student’s t-test). Annotation level (Ann. Level): 1. compound identified using a synthesized standard; 2. compound putatively annotated by interpretation of mass spectrometry data; 3. compound class putatively annotated; 4. unknown compounds. m/z: Mass to charge ratio. Annotation: MS2T identifiers, elemental composition of the uncharged compound, adducts or the identified compounds are given. Molecular Mass: Molecular mass of the respective compound (i.e. precursor or M) is given. (DOCX) [file pone.0102444.s006.docx]

**Supplemental File S6**

**Table S1: Markers consistently up-regulated upon hydroponic cultivation in Fe-free and alkaline medium condition in *A. thaliana* as detected by UPLC-ESI-QTOF-MS (positive ionization mode).** Markers from three biological replicates were chosen when they met the following criteria in both sets of three biologically independent experiments each: Fold-change>2 and P<0.05 (Student’s t-test). Annotation level (Ann. Level): 1, compound identified using a synthesized standard; 2, compound putatively annotated by interpretation of mass spectrometry data; 3, compound class putatively annotated; 4. unknown compounds. m/z: Mass to charge ratio. Annotation: MS2T identifiers, elemental composition of the uncharged compound, adducts or the identified compounds are given. Molecular Mass: Molecular mass of the respective compound (i.e. precursor or M) is given.

| Marker | Ann. Level | Retention Time (min) | m/z | Annotation | Molecular Mass | Fold-change 1/10 Hoagland´s solution without iron | Fold change alkaline 1/10 Hoagland´s Solution | Maximum Intensitiy 1/10 Hoagland´s solution without Iron | Maximum Intensitiy alkaline 1/10 Hoagland´s solution |
| --- | --- | --- | --- | --- | --- | --- | --- | --- | --- |
| p1 | 4 | 0.4 | 213.917 |  |  | 12.4 | 99.6 | 879 | 3607 |
| p2 | 4 | 0.4 | 198.941 |  |  | 2.2 | 2.8 | 2343 | 5448 |
| p3 | 4 | 0.55 | 268.947 |  |  | 2.1 | 2.1 | 2220 | 1519 |
| p4 | 4 | 0.87 | 84.081 |  |  | 5.8 | 3.5 | 4593 | 5021 |
| p5 | 4 | 0.87 | 130.086 |  |  | 2.6 | 2.7 | 13592 | 29487 |
| p6 | 4 | 1.05 | 407.031 |  |  | 3.6 | 8 | 209 | 311 |
| p7 | 4 | 1.05 | 129.019 |  |  | 2.8 | 6 | 2366 | 11359 |
| p8 | 3 | 1.05 | 409.019 | GMA01p07960. |  | 4.3 | 3.8 | 580 | 510 |
| p9 | 4 | 1.05 | 179.997 |  |  | 2.8 | 4.2 | 725 | 881 |
| p10 | 4 | 1.05 | 147.038 |  |  | 2.3 | 5.1 | 387 | 995 |
| p11 | 3 | 1.05 | 139.003 |  |  | 2.8 | 5.9 | 5003 | 15036 |
| p12 | 3 | 1.05 | 423.007 |  |  | 2.2 | 3.8 | 726 | 634 |
| p13 | 4 | 1.05 | 111.008 |  |  | 2.8 | 4.9 | 4131 | 4156 |
| p14 | 3 | 1.05 | 438.981 |  |  | 3 | 9.6 | 1769 | 2382 |
| p15 | 4 | 1.06 | 215.016 |  |  | 2.9 | 6.7 | 4514 | 12317 |
| p16 | 4 | 1.06 | 212.005 |  |  | 3.8 | 5.9 | 798 | 2828 |
| p17 | 4 | 2.88 | 211.06 |  |  | 24.3 | 51.2 | 606 | 1095 |
| p18 | 4 | 2.92 | 146.077 | fragment of p21  C6H11NO2 | 307.126 | 2.6 | 14.6 | 5750 | 21553 |
| p19 | 4 | 2.92 | 110.061 |  |  | 5.2 | 8.7 | 828 | 756 |
| p20 | 3 | 2.92 | 330.116 | OSA17p04889 [M+N. i.]+  p21 | 307.126 | 7.5 | 10.2 | 3412 | 5946 |
| p21 | 3 | 2.93 | 308.134 | ATH64p04805 C6H11NO2 -Hex | 307.126 | 6.1 | 11.6 | 945 | 9558 |
| p22 | 3 | 2.93 | 82.065 | fragment of p21 |  | 5.4 | 7.5 | 383 | 480 |
| p23 | 3 | 2.93 | 128.071 | fragment of p21 |  | 8.7 | 13.9 | 2319 | 4347 |
| p24 | 4 | 3.79 | 163.04 |  |  | 2.3 | 7.8 | 449 | 1495 |
| p25 | 1 | 3.95 | 193.05 | scopoletin fragment of scopolin |  | 2.3 | 5.2 | 28469 | 36320 |
| p26 | 3 | 3.95 | 225.04 | ATH64p07367  aglycon of  C10H8O6-Hex. | 386.088 | 208.3 | 9.7 | 43928 | 1965 |
| p27 | 1 | 4.12 | 393.079 | fraxin [M+N. i.]+ |  | 9 | 38.1 | 1122 | 5117 |
| p28 | 1 | 4.13 | 209.045 | fraxetin fragment of Fraxin |  | 13.7 | 466.3 | 1916 | 11125 |
| p29 | 4 | 4.24 | 436.143 |  |  | 6.7 | 13.4 | 696 | 1110 |
| p30 | 3 | 4.25 | 445.162 | [M+N. i.]+t | 422.173 | 2 | 5.8 | 886 | 1431 |
| p31 | 3 | 4.39 | 347.121 | of p32  [M+N. i.]+ | 324.130 | 2.5 | 5.7 | 890 | 1200 |
| p32 | 3 | 4.39 | 325.138 | OSA09p03259 | 324.130 | 2.5 | 5.2 | 742 | 783 |
| p33 | 4 | 4.41 | 407.095 |  |  | 27.3 | 116.3 | 619 | 1432 |
| p34 | 4 | 4.52 | 205.127 |  |  | 2.5 | 12.4 | 312 | 850 |
| p35 | 1 | 4.6 | 209.045 | fraxetin | 208.038 | 9.8 | 156.2 | 2613 | 6860 |
| p36 | 3 | 4.6 | 511.172 | C29H28O7  [M+N. i.]+ | 488.184 | 19.1 | 32.6 | 1410 | 1051 |
| p37 | 4 | 4.6 | 149.075 |  |  | 14.9 | 42.7 | 1754 | 1977 |
| p38 | 3 | 4.73 | 463.085 | C21H18O12 | 462.077 | 3.4 | 8.2 | 310 | 1460 |
| p39 | 3 | 4.88 | 206.082 | ATH14p02976. C10H7NO4 | 205.074 | 2.3 | 7.5 | 1151 | 3967 |
| p40 | 4 | 4.88 | 188.072 |  |  | 2.1 | 5 | 513 | 615 |
| p41 | 3 | 5.14 | 545.199 | C20H24O6-Hex  [M+N. i.]+ | 522.210 | 8.5 | 10.5 | 1567 | 1438 |
| p42 | 3 | 5.14 | 219.102 | ATH64p01665 fragment of p41 | 522.210 | 9.8 | 13.2 | 11420 | 11601 |
| p43 | 4 | 5.14 | 281.082 |  |  | 4.4 | 4.2 | 347 | 398 |
| p44 | 3 | 5.2 | 323.128 | fragment of p54 | 322.120 | 3 | 5.1 | 247 | 405 |
| p45 | 3 | 5.2 | 341.139 | ATH69p43024 C20H20O5 | 340.131 | 4.6 | 14.3 | 721 | 1510 |
| p46 | 3 | 5.2 | 543.184 | C20H22O6-Hex [M+N. i.]+ | 520.195 | 4.7 | 12.2 | 770 | 1314 |
| p47 | 3 | 5.22 | 193.05 | scopoletin |  | 3.1 | 6.1 | 10397 | 23544 |
| p48 | 4 | 5.63 | 299.111 |  |  | 3.2 | 9 | 267 | 347 |
| p49 | 3 | 6.01 | 595.193 | ATH66p11892  C28H34O14 | 594.085 | 4.8 | 126.7 | 2500 | 438 |
| p50 | 4 | 7.05 | 206.068 |  |  | 2.1 | 2.2 | 425 | 907 |
| p51 | 4 | 7.05 | 228.05 |  |  | 2.3 | 3.2 | 421 | 658 |
| p52 | 4 | 7.34 | 419.171 |  |  | 4.7 | 4.3 | 407 | 828 |
| p53 | 3 | 7.68 | 207.066 | similar to MassBank record PR101042. sin. i.pic aicd |  | 17.6 | 21.2 | 688 | 427 |

**Table S2: Markers consistently up-regulated upon hydroponic cultivation in Fe-free and alkaline medium condition in *A. thaliana* as detected by UPLC-ESI-QTOF-MS (negative mode).** Markers from three biological replicates were chosen when they met the following criteria in both sets of three biologically independent experiments each: Fold-change>2 and p-value <0.05 (Student’s t-test). Annotation level (Ann. Level): 1. compound identified using a synthesized standard; 2. compound putatively annotated by interpretation of mass spectrometry data; 3. compound class putatively annotated; 4. unknown compounds. m/z: Mass to charge ratio. Annotation: MS2T identifiers, elemental composition of the uncharged compound, adducts or the identified compounds are given. Molecular Mass: Molecular mass of the respective compound (i.e. precursor or M) is given.

| Marker | Ann. Level | Retention Time (min) | m/z | Annotation | Molecular Mass | Fold-change 1/10 Hoagland´s solution without iron | Fold change alkaline 1/10 Hoagland´s Solution | Maximum Intensitiy 1/10 Hoagland´s solution without Iron | Maximum Intensitiy alkaline 1/10 Hoagland´s solution |
| --- | --- | --- | --- | --- | --- | --- | --- | --- | --- |
| n1 | 4 | 1.07 | 129.019 |  |  | 2.8 | 4 | 517 | 746 |
| n2 | 4 | 2.43 | 131.034 |  |  | 6.2 | 4.7 | 659 | 972 |
| n3 | 3 | 2.69 | 315.072 | ATH09n04709 C7H6O5-Hex | 316.080 | 2.8 | 6.9 | 286 | 727 |
| n4 | 4 | 2.93 | 306.119 |  | M:307.126 | 7.2 | 15 | 411 | 1137 |
| n5 | 4 | 3.05 | 329.087 |  |  | 2.4 | 2.6 | 439 | 809 |
| n6 | 4 | 3.69 | 697.235 |  |  | 3.1 | 11.2 | 1158 | 2971 |
| n7 | 4 | 3.79 | 341.088 |  |  | 2.2 | 6.4 | 928 | 1787 |
| n8 | 1 | 3.95 | 191.034 | scopoletin fragment of scopolin | 2.7 | 3.6 | 3473 | 3827 |  |
| n9 | 1 | 3.95 | 399.093 | scopolin [M-H+HCOOH]- | 3.1 | 5.5 | 3306 | 11517 |  |
| n10 | 1 | 4.13 | 369.083 | fraxin | 370.092 | 47.5 | 315.4 | 453 | 3701 |
| n11 | 3 | 4.24 | 421.165 | C25H26O6 (422.1729) | 422.173 | 2.1 | 4.6 | 463 | 2266 |
| n12 | 4 | 4.24 | 277.122 |  |  | 2.1 | 3.8 | 728 | 1229 |
| n13 | 4 | 4.29 | 307.085 |  |  | 2.1 | 4.4 | 455 | 748 |
| n14 | 4 | 4.38 | 221.049 |  |  | 6.5 | 25.9 | 314 | 797 |
| n15 | 4 | 4.51 | 293.106 |  |  | 3.9 | 27.1 | 446 | 2404 |
| n16 | 3 | 4.6 | 277.122 | ATH06n01303 | 15 | 18.8 | 941 | 763 |  |
| n17 | 3 | 4.6 | 487.176 | ATH08n02983 | 488.184 | 23.8 | 44.1 | 1654 | 1689 |
| n18 | 1 | 4.6 | 207.029 | fraxetin | 208.038 | 2.3 | 11.6 | 905 | 3173 |
| n19 | 4 | 4.86 | 423.187 |  |  | 2.7 | 6.1 | 497 | 764 |
| n20 | 3 | 4.88 | 206.081 | ATH64n02157 C11H13NO3 | 207.089 | 2.6 | 7.4 | 1243 | 5690 |
| n21 | 4 | 4.88 | 385.114 |  |  | 2.1 | 2.8 | 540 | 809 |
| n22 | 4 | 4.96 | 487.146 |  | 488.153 | 2.1 | 3.8 | 418 | 623 |
| n23 | 3 | 4.96 | 443.155 | ATH14n06535 | 488.153 | 2.1 | 3.9 | 3300 | 5263 |
| n24 | 3 | 5.14 | 359.15 | ATH59n06075   C20H24O6 aglycone of n25 | 522.21 | 10.1 | 7.3 | 820 | 351 |
| n25 | 3 | 5.14 | 521.203 | ATH59n09791  C20H24O6-Hex | 522.21 | 11.5 | 10.4 | 7907 | 13683 |
| n26 | 4 | 5.14 | 567.208 | [M-H+HCOOH]- | 522.21 | 13.1 | 13.9 | 1410 | 3223 |
| n27 | 4 | 5.14 | 589.19 | [M-H+NaCOOH]- | 522.21 | 9.7 | 6.6 | 388 | 230 |
| n28 | 4 | 5.14 | 557.179 | [M+Cl]- | 522.21 | 9.2 | 8.2 | 233 | 292 |
| n29 | 3 | 5.19 | 519.187 | ATH14n07562 C20H22O6-Hex | 520.195 | 8.7 | 17.1 | 2339 | 5645 |
| n30 | 1 | 5.22 | 191.034 | scopoletin |  | 3.1 | 4.8 | 1342 | 2718 |
| n31 | 3 | 5.22 | 609.172 | C28H34O15 | 610.182 | 4.5 | 4.4 | 761 | 600 |
| n32 | 4 | 5.23 | 176.011 |  |  | 3.7 | 4.7 | 610 | 535 |
| n33 | 4 | 5.23 | 477.139 |  |  | 3.3 | 2.5 | 277 | 607 |
| n34 | 4 | 6.98 | 263.095 |  |  | 6.9 | 12.1 | 558 | 788 |
| n35 | 4 | 7.32 | 395.174 |  |  | 5.8 | 3.9 | 702 | 2312 |
| n36 | 3 | 7.68 | 727.26 | ATH14n11325 |  | 71.1 | 43.7 | 1522 | 1225 |
| n33 | 4 | 5.23 | 477.139 |  |  | 3.3 | 2.5 | 277 | 607 |
| n34 | 4 | 6.98 | 263.095 |  |  | 6.9 | 12.1 | 558 | 788 |
| n35 | 4 | 7.32 | 395.174 |  |  | 5.8 | 3.9 | 702 | 2312 |
| n36 | 3 | 7.68 | 727.26 | ATH14n11325 |  | 71.1 | 43.7 | 1522 | 1225 |
|  |  |  |  |  |  |  |  |  |  |
