# Supplementary material for: Metabolome Analysis of Arabidopsis thaliana Roots Identifies a Key Metabolic Pathway for Iron Acquisition
Source: PLoS One. 2014 Jul 24;9(7):e102444. doi: 10.1371/journal.pone.0102444 (PMC4109925; doi:10.1371/journal.pone.0102444)
Supplement: File S7 — Tables S3 and S4 listing markers identified in a comparison of root exudates harvested from A. thaliana wild type and f6′h1-5 mutant plants as detected by UPLC-ESI-QTOF-MS in either positive ionization mode (Table S3) or negative ionization mode (Table S4). Markers accumulating in the wild-type exudates were chosen when they met the following criteria: Fold-change >2 and P<0.05 (Student’s t-test; 3 independent biological experiments). Annotation level (Ann. Level): 1. compound identified using a synthesized standard; 2. compound putatively annotated by interpretation of mass spectrometry data; 3. compound class putatively annotated; 4. unknown compounds. m/z: Mass to charge ratio. Annotation: adducts or the identified compounds are given. Molecular Mass: Molecular mass of the respective compound (i.e. precursor or M) is given. Entries are ordered according to their fold-change. (DOCX) [file pone.0102444.s007.docx]

**Supplemental File S7**

**Table S3: Comparison of root exudates harvested from *A. thaliana* wild type and *f6´h1-1* mutant plants as detected by UPLC-ESI-QTOF-MS (positive ionization mode).** Markers accumulating in the wild-type exudates were chosen when they met the following criteria: Fold-change>2 and P<0.05 (Student’s t-test; 3 independent biological experiments). Annotation level (Ann. Level): 1. compound identified using a synthesized standard; 2. compound putatively annotated by interpretation of mass spectrometry data; 3. compound class putatively annotated; 4. unknown compounds. *m/z*: Mass to charge ratio. Annotation: adducts or the identified compounds are given. Molecular Mass: Molecular mass of the respective compound (i.e. precursor or M) is given. Entries are ordered according to their fold-change.

| Marker | Ann. Level | Retention Time (min) | m/z | Annotation | Molecular Mass | Fold-change | Intensitiy wt | Intensitiy f6´h1-1 |
| --- | --- | --- | --- | --- | --- | --- | --- | --- |
| ex_p1 | 1 | 6.22 | 258.098 |  | 257.09 | 39.3 | 3398 ± 2819 | 0 ± 80 |
| ex_p2 | 4 | 5.23 | 193.051 | scopoletin |  | 34.6 | 11597 ± 4100 | 0 ± 63 |
| ex_p3 | 4 | 5.23 | 145.521 | fragment of scolopetin |  | 29.9 | 486 ± 153 | 0 ± 4 |
| ex_p4 | 4 | 5.23 | 215.034 | [M+Na]+ of scopooletin |  | 25.9 | 448 ± 135 | 0 ± 6 |
| ex_p5 | 4 | 4.14 | 179.036 | esculetin |  | 17.8 | 879 ± 532 | 220 ± 7 |
| ex_p6 | 1 | 5.83 | 359.037 |  |  | 13.5 | 432 ± 261 | 0 ± 18 |
| ex_p7 | 1 | 5.61 | 391.155 |  |  | 12.7 | 1102 ± 220 | 0 ± 24 |
| ex_p8 | 1 | 6.23 | 300.037 |  |  | 12 | 1014 ± 643 | 0 ± 87 |
| ex_p9 | 1 | 6.22 | 298.04 |  |  | 9.9 | 1931 ± 1177 | 0 ± 136 |
| ex_p10 | 1 | 6.17 | 346.106 |  |  | 8.8 | 407 ± 116 | 0 ± 14 |
| ex_p11 | 1 | 2.59 | 168.049 |  |  | 8.2 | 1759 ± 599 | 183 ± 105 |
| ex_p12 | 1 | 5.23 | 157.029 |  |  | 8 | 869 ± 269 | 0 ± 15 |
| ex_p13 | 1 | 0.46 | 214.847 |  | 213.839 | 6.9 | 1133 ± 1027 | 203 ± 123 |
| ex_p14 | 1 | 6.4 | 279.091 |  | 278.083 | 6.8 | 6238 ± 3322 | 0 ± 600 |
| ex_p15 | 1 | 6.23 | 236.117 |  |  | 6.8 | 1402 ± 896 | 0 ± 135 |
| ex_p16 | 1 | 0.46 | 212.85 |  |  | 6.6 | 4034 ± 3642 | 230 ± 539 |
| ex_p17 | 1 | 5.61 | 431.095 |  |  | 6.5 | 371 ± 119 | 0 ± 24 |
| ex_p18 | 1 | 3.75 | 436.147 |  |  | 6.4 | 1360 ± 241 | 1347 ± 222 |
| ex_p19 | 1 | 3.76 | 538.086 |  |  | 6.3 | 613 ± 101 | 121 ± 96 |
| ex_p20 | 1 | 5.07 | 412.144 |  |  | 6 | 1188 ± 843 | 0 ± 170 |
| ex_p21 | 1 | 3.76 | 252.112 |  |  | 5.9 | 1210 ± 256 | 144 ± 190 |
| ex_p22 | 1 | 3.39 | 208.14 |  |  | 5.6 | 6191 ± 6196 | 84 ± 784 |
| ex_p23 | 1 | 3.76 | 184.118 |  |  | 5.6 | 873 ± 199 | 250 ± 139 |
| ex_p24 | 1 | 7.45 | 241.177 |  |  | 5 | 999 ± 473 | 0 ± 75 |
| ex_p25 | 1 | 7.29 | 447.218 |  | 446.21 | 4.9 | 2707 ± 1178 | 0 ± 121 |
| ex_p26 | 1 | 3.75 | 532.069 |  |  | 4.8 | 1126 ± 170 | 302 ± 229 |
| ex_p27 | 1 | 4.75 | 102.056 |  |  | 4.8 | 524 ± 326 | 0 ± 60 |
| ex_p28 | 1 | 7.25 | 369.174 |  |  | 4.8 | 577 ± 338 | 0 ± 37 |
| ex_p29 | 1 | 3.66 | 365.124 |  |  | 4.7 | 2738 ± 2857 | 157 ± 545 |
| ex_p30 | 1 | 4.97 | 130.068 |  |  | 4.6 | 2894 ± 1216 | 0 ± 434 |
| ex_p31 | 1 | 4.74 | 389.174 |  |  | 4.5 | 2582 ± 1367 | 401 ± 378 |
| ex_p32 | 1 | 6.95 | 240.106 |  |  | 4.5 | 1589 ± 996 | 0 ± 95 |
| ex_p33 | 1 | 5.09 | 234.101 |  |  | 4.4 | 1022 ± 627 | 0 ± 156 |
| ex_p34 | 1 | 4.74 | 177.09 |  |  | 4.3 | 697 ± 395 | 0 ± 105 |
| ex_p35 | 1 | 6.42 | 234.102 |  |  | 4.3 | 670 ± 159 | 0 ± 56 |
| ex_p36 | 1 | 3.76 | 202.128 |  |  | 4.2 | 511 ± 157 | 551 ± 86 |
| ex_p37 | 1 | 4.65 | 447.18 |  |  | 4.1 | 1045 ± 593 | 283 ± 164 |
| ex_p38 | 1 | 7.28 | 425.236 |  |  | 4 | 1106 ± 504 | 0 ± 76 |
| ex_p39 | 1 | 2.69 | 146.062 |  |  | 3.9 | 2273 ± 1378 | 2187 ± 500 |
| ex_p40 | 1 | 5.09 | 192.144 |  |  | 3.9 | 1772 ± 939 | 0 ± 246 |
| ex_p41 | 1 | 5.25 | 207.068 |  |  | 3.9 | 792 ± 475 | 0 ± 147 |
| ex_p42 | 1 | 3.42 | 224.111 |  |  | 3.8 | 1797 ± 529 | 205 ± 273 |
| ex_p43 | 1 | 4.22 | 321.132 |  |  | 3.8 | 1046 ± 546 | 456 ± 114 |
| ex_p44 | 1 | 4.74 | 283.136 |  |  | 3.8 | 1532 ± 757 | 724 ± 208 |
| ex_p45 | 1 | 4.95 | 230.121 |  |  | 3.8 | 451 ± 213 | 0 ± 55 |
| ex_p46 | 1 | 5.04 | 213.095 |  |  | 3.8 | 2850 ± 1686 | 0 ± 272 |
| ex_p47 | 1 | 6.82 | 145.054 |  |  | 3.8 | 551 ± 146 | 0 ± 83 |
| ex_p48 | 1 | 3.75 | 224.11 |  |  | 3.7 | 4061 ± 530 | 350 ± 936 |
| ex_p49 | 1 | 4.75 | 365.088 |  |  | 3.6 | 512 ± 102 | 0 ± 115 |
| ex_p50 | 1 | 6.1 | 202.134 |  | 201.126 | 3.6 | 7937 ± 3662 | 0 ± 517 |
| ex_p51 | 1 | 6.1 | 224.114 |  | 223.106 | 3.6 | 46801 ± 17520 | 0 ± 2505 |
| ex_p52 | 1 | 5.16 | 200.022 |  |  | 3.5 | 763 ± 384 | 0 ± 130 |
| ex_p53 | 1 | 3.35 | 182.064 |  |  | 3.4 | 1021 ± 299 | 917 ± 227 |
| ex_p54 | 1 | 5.88 | 242.127 |  | 241.119 | 3.4 | 5866 ± 4171 | 0 ± 1089 |
| ex_p55 | 1 | 2.06 | 163.986 |  |  | 3.3 | 1547 ± 1040 | 87 ± 427 |
| ex_p56 | 1 | 3.55 | 130.067 |  |  | 3.3 | 1587 ± 709 | 499 ± 354 |
| ex_p57 | 1 | 6.23 | 420.152 |  |  | 3.3 | 1644 ± 473 | 0 ± 368 |
| ex_p58 | 1 | 0.53 | 236.15 |  |  | 3.2 | 4960 ± 1084 | 343 ± 1105 |
| ex_p59 | 1 | 6.11 | 138.13 |  |  | 3.2 | 1453 ± 658 | #BEZUG! |
| ex_p60 | 1 | 7.31 | 192.068 |  |  | 3.2 | 1137 ± 517 | 0 ± 194 |
| ex_p61 | 1 | 7.31 | 148.078 |  |  | 3.2 | 692 ± 316 | 0 ± 123 |
| ex_p62 | 1 | 0.56 | 302.197 |  | 301.189 | 3.1 | 4383 ± 3092 | 16 ± 964 |
| ex_p63 | 1 | 5.25 | 175.042 |  |  | 3.1 | 318 ± 186 | 0 ± 68 |
| ex_p64 | 1 | 5.34 | 541.185 |  |  | 3.1 | 894 ± 691 | 0 ± 162 |
| ex_p65 | 1 | 6.04 | 227.111 |  |  | 3.1 | 2590 ± 1779 | 0 ± 417 |
| ex_p66 | 1 | 6.23 | 516.074 |  |  | 3.1 | 719 ± 156 | 0 ± 162 |
| ex_p67 | 1 | 6.82 | 160.078 |  |  | 3.1 | 930 ± 236 | 0 ± 158 |
| ex_p68 | 1 | 2.34 | 103.06 |  |  | 3 | 4929 ± 2619 | 718 ± 1203 |
| ex_p69 | 1 | 2.72 | 130.067 |  |  | 3 | 4350 ± 3104 | 457 ± 1599 |
| ex_p70 | 1 | 6.23 | 522.091 |  |  | 3 | 1156 ± 255 | 0 ± 252 |
| ex_p71 | 1 | 2.38 | 163.986 |  |  | 2.9 | 2240 ± 676 | 57 ± 479 |
| ex_p72 | 1 | 5.14 | 188.113 |  |  | 2.9 | 1001 ± 415 | 0 ± 164 |
| ex_p73 | 1 | 2.41 | 137.049 |  |  | 2.8 | 391 ± 229 | 1717 ± 153 |
| ex_p74 | 1 | 4.03 | 442.085 |  |  | 2.8 | 3580 ± 2414 | 287 ± 869 |
| ex_p75 | 1 | 5.08 | 256.083 |  |  | 2.8 | 561 ± 254 | 0 ± 120 |
| ex_p76 | 1 | 5.14 | 210.095 |  | 209.087 | 2.8 | 6740 ± 2671 | 0 ± 1239 |
| ex_p77 | 1 | 5.94 | 441.232 |  |  | 2.8 | 516 ± 188 | 0 ± 84 |
| ex_p78 | 1 | 0.61 | 192.145 |  |  | 2.7 | 2548 ± 540 | 109 ± 496 |
| ex_p79 | 1 | 2.88 | 302.199 |  |  | 2.7 | 2811 ± 2090 | 828 ± 734 |
| ex_p80 | 1 | 6.07 | 272.132 |  |  | 2.7 | 1017 ± 601 | 0 ± 215 |
| ex_p81 | 1 | 0.53 | 192.161 |  |  | 2.6 | 1362 ± 939 | 2428 ± 425 |
| ex_p82 | 1 | 0.91 | 124.041 |  |  | 2.6 | 4223 ± 717 | 335 ± 967 |
| ex_p83 | 1 | 1.02 | 123.057 |  |  | 2.6 | 5328 ± 4853 | 17 ± 3035 |
| ex_p84 | 1 | 4.03 | 464.068 |  |  | 2.6 | 1368 ± 901 | 350 ± 332 |
| ex_p85 | 1 | 4.9 | 307.118 |  | 306.11 | 2.6 | 1770 ± 1303 | 0 ± 455 |
| ex_p86 | 1 | 7 | 305.137 |  | 304.129 | 2.6 | 3561 ± 1711 | 0 ± 693 |
| ex_p87 | 1 | 7.87 | 490.122 |  |  | 2.6 | 747 ± 505 | 0 ± 170 |
| ex_p88 | 1 | 0.51 | 332.968 |  |  | 2.5 | 958 ± 822 | 456 ± 446 |
| ex_p89 | 1 | 3.11 | 130.067 |  |  | 2.5 | 24065 ± 14183 | 13142 ± 6581 |
| ex_p90 | 1 | 5.42 | 272.132 |  |  | 2.5 | 514 ± 223 | 0 ± 61 |
| ex_p91 | 1 | 7.03 | 238.126 |  |  | 2.5 | 621 ± 395 | 0 ± 84 |
| ex_p92 | 1 | 1.59 | 202.183 |  | 201.175 | 2.4 | 15949 ± 11428 | 286 ± 6518 |
| ex_p93 | 1 | 3.36 | 258.173 |  |  | 2.4 | 2038 ± 1607 | 86 ± 652 |
| ex_p94 | 1 | 4.01 | 160.078 |  | 159.07 | 2.4 | 16991 ± 9007 | 489 ± 5493 |
| ex_p95 | 1 | 6.1 | 160.113 |  |  | 2.4 | 915 ± 302 | 0 ± 87 |
| ex_p96 | 1 | 7.97 | 339.126 |  |  | 2.4 | 1721 ± 811 | 0 ± 522 |
| ex_p97 | 1 | 1.6 | 102.093 |  |  | 2.3 | 702 ± 489 | 208 ± 256 |
| ex_p98 | 1 | 3.41 | 298.099 |  |  | 2.3 | 697 ± 284 | 195 ± 232 |
| ex_p99 | 1 | 3.54 | 114.092 |  | 113.084 | 2.3 | 6336 ± 2381 | 234 ± 293 |
| ex_p100 | 1 | 3.65 | 190.052 |  |  | 2.3 | 381 ± 220 | 277 ± 106 |
| ex_p101 | 1 | 4.01 | 132.083 |  |  | 2.3 | 1213 ± 593 | 276 ± 339 |
| ex_p102 | 1 | 7.97 | 322.1 |  |  | 2.3 | 928 ± 434 | 0 ± 289 |
| ex_p103 | 1 | 0.95 | 192.157 |  |  | 2.2 | 1028 ± 487 | 206 ± 266 |
| ex_p104 | 1 | 2.37 | 117.98 |  |  | 2.2 | 1670 ± 618 | 32 ± 237 |
| ex_p105 | 1 | 3.27 | 302.198 |  | 301.19 | 2.2 | 5600 ± 4078 | 46 ± 1923 |
| ex_p106 | 1 | 3.67 | 138.057 |  | 137.049 | 2.2 | 10112 ± 5747 | 380 ± 3253 |
| ex_p107 | 1 | 5.83 | 160.079 |  |  | 2.2 | 1595 ± 245 | 0 ± 337 |
| ex_p108 | 1 | 6.57 | 248.172 |  |  | 2.2 | 598 ± 236 | 0 ± 175 |
| ex_p109 | 1 | 7.29 | 489.157 |  |  | 2.2 | 643 ± 112 | 0 ± 65 |
| ex_p110 | 1 | 7.71 | 216.199 |  |  | 2.2 | 981 ± 571 | 0 ± 244 |
| ex_p111 | 1 | 0.56 | 124.049 |  |  | 2.1 | 1892 ± 550 | 217 ± 491 |
| ex_p112 | 1 | 1.37 | 158.157 |  |  | 2.1 | 2342 ± 1618 | 102 ± 958 |
| ex_p113 | 1 | 2.93 | 214.126 |  |  | 2.1 | 4098 ± 1306 | 372 ± 719 |
| ex_p114 | 1 | 4.56 | 130.067 |  |  | 2.1 | 843 ± 474 | 816 ± 250 |
| ex_p115 | 1 | 7.29 | 487.159 |  |  | 2.1 | 1033 ± 172 | 0 ± 48 |
| ex_p116 | 1 | 7.71 | 170.193 |  |  | 2.1 | 1716 ± 990 | 0 ± 468 |
| ex_p117 | 1 | 4.2 | 196.08 |  |  | 2 | 3951 ± 1959 | 201 ± 1286 |

**Table S4: Comparison of root exudates harvested from *A. thaliana* wild type and *f6´h1-1* mutant plants as detected by UPLC-ESI-QTOF-MS (negative ionization mode).** Markers accumulating in the wild-type exudates were chosen when they met the following criteria: Fold-change>2 and p-value <0.05 (Student’s t-test; 3 independent biological experiments). Annotation level (Ann. Level): 1. compound identified using a synthesized standard; 2. compound putatively annotated by interpretation of mass spectrometry data; 3. compound class putatively annotated; 4. unknown compounds. *m/z*: Mass to charge ratio. Annotation: adducts or the identified compounds are given. Molecular Mass: Molecular mass of the respective compound (i.e. precursor or M) is given. Entries are ordered according to their fold-change.

| Marker | Ann. Level | Retention Time (min) | m/z | Annotation | Molecular Mass | Fold-change | Intensitiy wt | Intensitiy f6´h1-1 |
| --- | --- | --- | --- | --- | --- | --- | --- | --- |
| ex_n1 | 1 | 5.22 | 191.035 | Scopoletin |  | 86.8 | 1798 ± 628 | 21 ± 6 |
| ex_n2 | 1 | 4.14 | 177.02 | Esculetin |  | 22 | 1144 ± 717 | 52 ± 12 |
| ex_n3 | 1 | 5.22 | 176.012 | fragment of scopoletin |  | 14.1 | 807 ± 295 | 57 ± 9 |
| ex_n4 | 4 | 0.49 | 232.898 |  |  | 9.8 | 1798 ± 738 | 183 ± 129 |
| ex_n5 | 4 | 15.9 | 733.536 | [M+Cl+NaCOOH]- | 630.577 | 9.5 | 1484 ± 1583 | 156 ± 159 |
| ex_n6 | 4 | 15.9 | 665.549 | [M+Cl]- | 630.577 | 9.5 | 2620 ± 2850 | 277 ± 282 |
| ex_n8 | 4 | 11.28 | 436.284 | [M+Cl]- | 401.314 | 9.2 | 1144 ± 478 | 124 ± 89 |
| ex_n7 | 4 | 12.23 | 546.284 | [M+Cl+NaCOOH]- | 443.324 | 9.2 | 888 ± 572 | 96 ± 34 |
| ex_n9 | 4 | 15.9 | 801.524 | [M+Cl+NaCOOH]- |  | 9.1 | 962 ± 1012 | 106 ± 103 |
| ex_n11 | 4 | 11.88 | 520.268 | [M+Cl+NaCOOH]- | 417.309 | 8.7 | 458 ± 114 | 53 ± 39 |
| ex_n10 | 4 | 12.23 | 478.296 | [M+Cl]- 443.324 | 443.324 | 8.7 | 2394 ± 1554 | 274 ± 94 |
| ex_n12 | 4 | 11.29 | 504.272 | [M+Cl+NaCOOH]- | 401.314 | 8.1 | 425 ± 179 | 53 ± 37 |
| ex_n13 | 4 | 20.9 | 295.076 |  |  | 7.6 | 1753 ± 1045 | 231 ± 146 |
| ex_n14 | 4 | 11.86 | 467.28 |  |  | 7.5 | 347 ± 167 | 46 ± 35 |
| ex_n15 | 4 | 11.88 | 452.28 | [M+Cl]- | 417.309 | 7.4 | 1227 ± 298 | 165 ± 89 |
| ex_n16 | 4 | 11.36 | 524.338 |  |  | 7.2 | 337 ± 177 | 47 ± 46 |
| ex_n17 | 4 | 20.37 | 551.197 | [M-2H+K] | 514.235 | 6.9 | 600 ± 285 | 87 ± 87 |
| ex_n18 | 4 | 20.37 | 549.204 | [M+Cl]- | 514.235 | 6.8 | 560 ± 277 | 83 ± 78 |
| ex_n19 | 4 | 21.19 | 295.077 |  |  | 6.6 | 689 ± 392 | 105 ± 59 |
| ex_n20 | 4 | 9.02 | 351.163 |  |  | 6.4 | 491 ± 132 | 76 ± 41 |
| ex_n21 | 4 | 0.54 | 436.043 |  |  | 6.2 | 2299 ± 1246 | 368 ± 483 |
| ex_n22 | 4 | 9.32 | 671.31 |  |  | 6.2 | 673 ± 106 | 109 ± 74 |
| ex_n23 | 4 | 11.02 | 518.252 | [M+Cl+NaCOOH]- | 415.293 | 6.1 | 821 ± 292 | 134 ± 76 |
| ex_n26 | 4 | 0.51 | 194.944 |  |  | 6 | 636 ± 70 | 107 ± 84 |
| ex_n25 | 4 | 3.45 | 492.108 |  | 491.1 | 6 | 2215 ± 702 | 368 ± 350 |
| ex_n24 | 4 | 11.02 | 450.266 | [M+Cl]- | 415.293 | 6 | 2362 ± 893 | 392 ± 236 |
| ex_n27 | 4 | 20.06 | 754.466 | [M+Cl+NaCOOH]- | 651.506 | 5.9 | 2284 ± 485 | 386 ± 210 |
| ex_n28 | 4 | 21.08 | 297.083 |  |  | 5.9 | 1407 ± 1021 | 239 ± 87 |
| ex_n29 | 4 | 3.77 | 560.094 | [M+Cl+NaCOOH]- | 457.134 | 5.4 | 968 ± 190 | 178 ± 176 |
| ex_n30 | 4 | 3.76 | 492.106 | [M+Cl]- | 457.134 | 5.3 | 8801 ± 1918 | 1649 ± 1681 |
| ex_n31 | 4 | 20.06 | 686.478 | [M+Cl]- | 651.506 | 5.2 | 5119 ± 1234 | 990 ± 462 |
| ex_n33 | 4 | 3.23 | 478.09 |  |  | 5.1 | 547 ± 217 | 108 ± 110 |
| ex_n32 | 4 | 4.07 | 401.146 |  |  | 5.1 | 788 ± 302 | 155 ± 113 |
| ex_n34 | 4 | 0.51 | 277.031 |  |  | 4.3 | 1313 ± 912 | 306 ± 419 |
| ex_n35 | 4 | 3.33 | 420.048 |  |  | 4.2 | 2222 ± 1913 | 526 ± 609 |
| ex_n36 | 4 | 5.48 | 462.095 |  |  | 3.7 | 1497 ± 530 | 402 ± 318 |
| ex_n37 | 4 | 6.23 | 476.112 | [M+Cl]- | 441.139 | 3.4 | 10657 ± 3371 | 3146 ± 2363 |
| ex_n39 | 4 | 3.56 | 447.055 |  | 450.587 | 3.3 | 2529 ± 470 | 773 ± 734 |
| ex_n38 | 4 | 21.84 | 591.432 |  |  | 3.3 | 831 ± 316 | 251 ± 30 |
| ex_n40 | 4 | 6.23 | 544.098 | [M+Cl+NaCOOH]- | 441.139 | 3.1 | 1696 ± 458 | 540 ± 403 |
| ex_n41 | 4 | 6.23 | 612.086 | [M+Cl+NaCOOH]- | 509.127 | 2.9 | 366 ± 75 | 128 ± 91 |
| ex_n43 | 4 | 4.74 | 440.069 |  |  | 2.6 | 854 ± 547 | 324 ± 208 |
| ex_n42 | 4 | 20.43 | 744.567 |  |  | 2.6 | 450 ± 284 | 171 ± 123 |
| ex_n44 | 4 | 0.51 | 96.965 |  |  | 2.5 | 7559 ± 1852 | 3002 ± 1305 |
| ex_n46 | 4 | 4.71 | 165.057 |  |  | 2.4 | 563 ± 169 | 232 ± 164 |
| ex_n47 | 4 | 4.73 | 477.067 |  |  | 2.4 | 1691 ± 380 | 709 ± 415 |
| ex_n45 | 4 | 21.18 | 465.286 |  |  | 2.4 | 884 ± 304 | 365 ± 82 |
| ex_n48 | 4 | 7.87 | 488.107 |  |  | 2.3 | 1115 ± 765 | 490 ± 284 |
| ex_n52 | 4 | 0.44 | 380.846 |  |  | 2.2 | 1387 ± 165 | 632 ± 598 |
| ex_n49 | 4 | 0.44 | 296.884 |  |  | 2.2 | 1759 ± 162 | 787 ± 696 |
| ex_n51 | 4 | 0.51 | 165.041 |  |  | 2.2 | 961 ± 218 | 432 ± 269 |
| ex_n50 | 4 | 4.03 | 440.07 |  | 443.547 | 2.2 | 15867 ± 10578 | 7117 ± 4786 |
| ex_n54 | 4 | 0.52 | 179.057 |  |  | 2 | 1464 ± 706 | 727 ± 706 |
| ex_n53 | 4 | 4.14 | 477.066 |  |  | 2 | 1823 ± 624 | 898 ± 531 |
